# Genetic variants and traits related to insulin-like growth factor-I and insulin resistance and their interaction with lifestyles on postmenopausal colorectal cancer risk

**DOI:** 10.1371/journal.pone.0186296

**Published:** 2017-10-12

**Authors:** Su Yon Jung, Thomas Rohan, Howard Strickler, Jennifer Bea, Zuo-Feng Zhang, Gloria Ho, Carolyn Crandall

**Affiliations:** 1 Translational Sciences Section, Jonsson Comprehensive Cancer Center, School of Nursing, University of California, Los Angeles, Los Angeles, California, United States of America; 2 Department of Epidemiology & Population Health, Albert Einstein College of Medicine, Bronx, New York, United States of America; 3 Medicine & Nutritional Sciences, University of Arizona Cancer Center, Tucson, Arizona, United States of America; 4 Department of Epidemiology, Fielding School of Public Health, University of California, Los Angeles, Los Angeles, California, United States of America; 5 Department of Occupational Medicine, Epidemiology and Prevention, Feinstein Institute for Medical Research, Hofstra Northwell School of Medicine, Great Neck, New York, United States of America; 6 Division of General Internal Medicine, Department of Internal Medicine, David Geffen School of Medicine, University of California, Los Angeles, Los Angeles, California, United States of America; University of South Alabama Mitchell Cancer Institute, UNITED STATES

## Abstract

Genetic variants and traits in metabolic signaling pathways may interact with lifestyle factors such as obesity, physical activity, and exogenous estrogen (E), influencing postmenopausal colorectal cancer (CRC) risk, but these interrelated pathways are not fully understood. In this case-cohort study, we examined 33 single-nucleotide polymorphisms (SNPs) in genes related to insulin-like growth factor-I (IGF-I)/ insulin resistance (IR) traits and signaling pathways, using data from 704 postmenopausal women in Women’s Health Initiative Observation ancillary studies. Stratifying by the lifestyle modifiers, we assessed the effects of IGF-I/IR traits (fasting total and free IGF-I, IGF binding protein-3, insulin, glucose, and homeostatic model assessment–insulin resistance) on CRC risk as a mediator or influencing factor. Six SNPs in the *INS*, *IGF-I*, and *IGFBP3* genes were associated with CRC risk, and those associations differed between non-obese/active and obese/inactive women and between E nonusers and users. Roughly 30% of the cancer risk due to the SNP was mediated by IGF-I/IR traits. Likewise, carriers of 11 SNPs in the *IRS1* and *AKT1/2* genes (signaling pathway–related genetic variants) had different associations with CRC risk between strata, and the proportion of the SNP–cancer association explained by traits varied from 30% to 50%. Our findings suggest that IGF-I/IR genetic variants interact with obesity, physical activity, and exogenous E, altering postmenopausal CRC risk, through IGF-I/IR traits, but also through different pathways. Unraveling gene–phenotype–lifestyle interactions will provide data on potential genetic targets in clinical trials for cancer prevention and intervention strategies to reduce CRC risk.

## Introduction

Colorectal cancer (CRC) is the third most commonly diagnosed cancer and the third leading cause of cancer death in American women.[1] Incidence and death rates for CRC increase with age. Approximately 90% of new cases and deaths occur in people of age 50 and older.[1] About 35% of the susceptibility to CRC is ascribed to genetic factors, while the remaining 65% is attributed to environmental factors such as obesity, physical activity, diet, smoking, and Type II diabetes (DM).[2] The effect of the lifestyle factors on CRC risk may be partially mediated by insulin-like growth factor-I (IGF-I)/insulin pathway. The IGF-I/insulin resistance (IR) axis has been associated with CRC in multiple studies.[3–5] Higher levels of total/ free bioactive IGF-I and lower levels of IGF-binding protein 3 (IGFBP3) have been associated with higher CRC risk in both pre- and post-menopausal women.[3, 4, 6]. In postmenopausal women, high levels of both insulin and glucose are positively associated with CRC.[4, 7]

High IGF-I levels and IR (characterized by hyperinsulinemia and hyperglycemia) contribute to overexpression of IGF/insulin receptors. The overexpression leads to the enhanced anabolic state necessary for cell proliferation, differentiation, and anti-apoptosis via multiple abnormal cellular signaling cascades, including insulin receptor substrate-1 (IRS-1) and protein kinase B (Akt) pathway.[8, 9] Thus, high IGF-I and IR, through deregulating or overactivating multiple downstream pathways, may exert their effects on carcinogenesis.

IGF-I/IR traits that have been associated with CRC risk include IGF-I, IGFBP3, insulin, glucose, and homeostatic model assessment–insulin resistance [HOMA-IR] levels in this study. Considering the associations of the IGF-I/IR traits and their signaling pathways with CRC risk, the genetic variants that may influence levels of the traits and aberrant signaling cascades are possibly associated with the risk of CRC. However, population-based epidemiologic studies of these genetic variants (e.g., single-nucleotide polymorphisms [SNPs]) and CRC risk have yielded inconsistent findings.[8, 10–17] These conflicts are possibly due to different sets of covariates (e.g., in women, whether to account for menopausal status or whether to consider different combinations of hormone therapy), lack of consideration for adjustment of relevant phenotypes and of interactions with lifestyle factors, and different races/ethnicities. Further, few studies examined those IGF-I/IR-related genetic variants and risk of CRC in postmenopausal women, a population highly susceptible to CRC.

Abdominal obesity and physical inactivity are associated with increased risk of postmenopausal CRC.[18–20] In particular, physical inactivity accounts for about 15% of CRC [21] and the highest physical activity (PA) guidelines scores, compared with the lowest scores, are associated with a 50% lower risk of CRC.[20] The relationships of obesity and obesity-related factors such as PA with CRC could be mediated via IGF-I/IR traits.[19, 21, 22] Few studies have examined whether the association between obesity/obesity factors and CRC risk is affected by IGF-I/IR genetic variants.[17, 21, 23]; though the genetic variants have minimal or modest effect on the obesity–CRC relationship, it suggests that genetic variants related to IGF-I/IR traits and their signaling pathways interact with obesity and jointly influence CRC susceptibility.

In addition, endogenous estrogen (E) interacts with IGF-I/IR traits and their signaling pathways as well as their target genes through a synergistic cross-talk mechanism, leading to an enhanced anabolic state necessary for tumor growth.[24–28] In postmenopausal women, endogenous E level is associated with higher risk of CRC.[4] Exogenous E has a different effect than endogenous E on CRC risk. Oral exogenous E has been postulated to interact with circulating levels of IGF-I/IR and their downstream pathways to affect cancer risk. Because of the first-pass effect induced by oral E, resulting in suppressing the production of IGF-I in liver, oral E users have lower IGF-I levels, followed by increased IR [29], and lower risk of CRC than E nonusers have. However, exogenous E has conflicting associations with CRC risk, depending on the combination of hormone therapy (E only vs. E + Progestin [P]) administered. While E + P users have consistently been related to a decreased CRC risk,[30, 31] E only users have not been uniformly associated with CRC risk: lower [32, 33] and higher [34] risks of CRC and nonsignificant associations [35, 36] have been observed.

By conducting this case-cohort study among non-Hispanic white postmenopausal women, we examined the pathway of IGF-I/IR traits/signaling–genetic variants, IGF-I/IR traits, and CRC risk. In this pathway, IGF-I/IR traits (circulating levels of IGF-I, IGFBP3, insulin, glucose, and HOMA-IR) have two different roles in the relationship between the genetic variants and CRC: mediator (in relation to IGF-I/IR traits–related genetic variants) and influencing factor (in relation to IGF-I/IR signaling pathways–related genetic variants).

Further, obesity status, PA, and exogenous E use status could influence the association between IGF-I/IR genetic factors and their traits, and through these interactions, are associated with CRC. We thus evaluated how the pathway of IGF-I/IR’s genetic variants, IGF-I/IR traits, and CRC is influenced by obesity, PA, and exogenous E (E only and E + P). Unraveling these complicated gene–phenotype–cancer pathways and interactions with lifestyle factors will provide insights into the role of the IGF-I/IR axis in the development of CRC in postmenopausal women.

## Materials and methods

### Study population

The study included 704 postmenopausal women who were enrolled in ancillary studies of the Women's Health Initiative Observational Study (WHI-OS) from October 1, 1993 through December 31, 1998. Details of the WHI’s design and rationale have been described elsewhere.[37] Eligible women were 50–79 years old, postmenopausal, planned to live near the clinical centers for at least 3 years after study enrollment, and able to provide written consent. The ancillary studies were designed for a nested case-cohort study within the WHI-OS, including only women who reported their race or ethnicity as non-Hispanic white (n = 2,148). For our study purpose, we initially included 1,136 of those women who were eligible for the colorectal case-cohort study (S1 Fig). Of those, we excluded 193 women who had been followed up for less than 1 year or had been diagnosed with any cancer at enrollment. Among these (n = 943), we included women (n = 887) who did not have DM at enrollment or later and had at least one of five measurements (i.e., total and free IGF-I, IGFBP3, glucose, and insulin obtained after at least 8 hours’ fast) available at baseline. We excluded another 2 women whose information on SNPs was not available or whose missing-call rates were more than 50%. Finally, we excluded 181 women for whom the information on covariates was unavailable, resulting in a total of 704 women (CRC cases = 237, controls = 467; 80% of the eligible 885). As of February 29, 2004, the ancillary studies completed the selection of women with a mean follow-up of 77 months.[38] This study was approved by the institutional review boards of each participating clinical center of the WHI and the University of California, Los Angeles.

### Data collection and cancer outcome variables

Data had been uniformly collected using standardized written protocols. At baseline, self-administered questionnaires were completed by participants regarding demographic factors (age, education, family income, and family histories of DM or CRC), lifestyle factors (PA, smoking status, and alcohol intake), and medical (cardiovascular disease and hypercholesterolemia) and reproductive histories (oral contraceptive and exogenous E use [never vs. ever use of unopposed estrogen (E only) and opposed estrogen (E + P) from pills or patches], history of hysterectomy or oophorectomy, ages at menopause and menarche, and pregnancy history). Anthropometric measurements such as height, weight, and waist and hip circumferences were measured at baseline by trained staff. The above variables were initially selected for this study on the basis of a literature review for their associations with IGF-I/IR and CRC. After multicollinearity testing and univariate and stepwise regression analyses, they were finally selected to be analyzed.

Cancer outcomes were determined through a centralized review of medical charts, and cancer cases were coded according to the National Cancer Institute’s Surveillance, Epidemiology, and End-Results guidelines.[39] The outcome variables for our study were CRC and the time to development of CRC. The time from enrollment to CRC development, censoring, or study end-point was recorded as the number of days and then converted into years.

### Genotyping and laboratory methods

Six genes (S1–S6 Tables) were chosen on the basis of the biologic significance of their gene products or whether epidemiologic and/or experimental data support an association between the gene and the levels of IGFs and insulin or between the gene and risk of cancer.[8, 40–50] For each gene, HTSNP2 software (http://www-gene.cimr.cam.ac.uk/clayton/software/stata) was used to search all possible subsets of SNPs that best captured the full haplotype information. Specifically, the selected SNPs had a minimum allelic association of 0.8 with the unselected SNPs within a linkage disequilibrium block. A total of 33 SNPs from the 6 genes were identified.

The MassARRAY system (Sequenom, Inc., San Diego, CA), based on mass spectrometry, was used for genotyping. Using a standardized protocol, quality assurance was conducted with a missing call rate of < 1%, the number of discordant calls < 3%, and a Hardy-Weinberg Equilibrium of p ≥ 10^−4^.

At baseline, fasting blood samples had been collected from each participant by trained phlebotomists. Serum concentrations of glucose and insulin were measured by Medical Research Laboratories (Highland Heights, KY) using assays with sensitivities of 0.5 mg/dL and 0.26 μIU/mL; average coefficients of variation (CV) of 4.2% and 3.4%; and correlation coefficients of 0.95 and 0.98, respectively. The HOMA-IR was calculated as glucose (mg/dl) × insulin (μIU/ml) / 405.[51] Serum total and free IGF-I and IGFBP3 were measured by using enzyme-linked immunosorbent assays (Diagnostic Systems Laboratories, Webster, TX) with sensitivities of 0.01 ng/mL, 0.015 ng/mL, and 0.04 ng/mL; average CVs of 8.2%, 11.2%, and 3.6%; and correlation coefficients of 0.96, 0.9, and 0.9, respectively.

### Statistical analysis

Differences in baseline characteristics and allele frequencies, across strata of obesity status (body mass index [BMI], waist circumference, and waist-to-hip ratio [w/h]), level of PA, and exogenous E use, were evaluated by using unpaired two-sample *t* tests for continuous variables and chi-squared tests for categorical variables. If continuous variables were skewed or had outliers, Wilcoxon’s rank-sum test was used.

With the regression assumptions met, multiple linear regression was performed to estimate effect sizes and 95% confidence intervals (CIs) for the exposures (IGF-I/IR–related SNPs with additive, minor-allele dominant and recessive models) to predict the outcomes (IGF-I/IR traits: fasting total and free IGF-I, IGFBP3, glucose, insulin, and HOMA-IR levels). The Cox proportional hazards regression model designed for case-cohort data was performed by using “cch” in a package “Survival” from the Comprehensive R Archive Network. After assumption testing was done via a Schoenfeld residual plot and rho, the Cox model was conducted to obtain hazard ratios (HRs) and 95% CIs for IGF-I/IR traits and IGF-I/IR–related SNPs to predict CRC.

**Fig 1:**
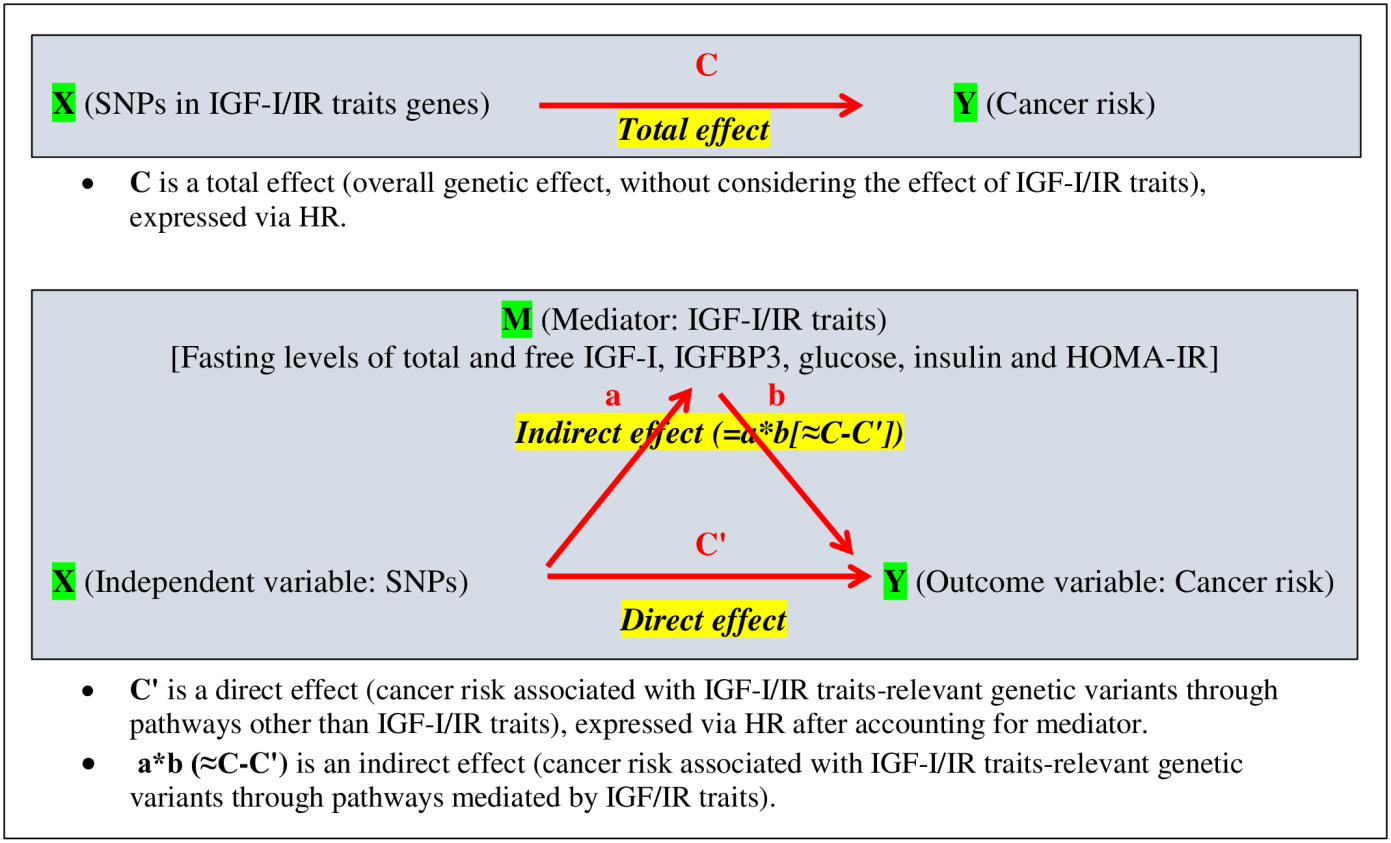
Diagrams of total, direct, and indirect pathways of SNPs in IGF-I/IR *traits* genes, IGF-I/IR traits, and colorectal cancer risk. (HOMA-IR, homeostatic model assessment–insulin resistance; HR, hazard ratio; IGF-I, insulin-like growth factor-I; IGFBP3, IGF binding protein 3; IR, insulin resistance; SNP, single-nucleotide polymorphism.)**C** is a total effect (overall genetic effect, without considering the effect of IGF-I/IR traits), expressed via HR. **C'** is a direct effect (cancer risk associated with IGF-I/IR traits-relevant genetic variants through pathways other than IGF-I/IR traits), expressed via HR after accounting for mediator. a*b (≈C-C') is an indirect effect (cancer risk associated with IGF-I/IR traits-relevant genetic variants through pathways mediated by IGF/IR traits).

We first focused on the mediation effects relating IGF-I/IR traits–related SNPs (exposure) and CRC (outcome), and on the role of IGF-I/IR traits (mediator) that play in this association (Fig 1). According to the models presented in Fig 1, we first obtained the magnitude of the total effect of IGF-I/IR traits–related SNPs on CRC (the overall genetic effect, without considering the effect of IGF-I/IR traits). We then evaluated how this total effect is partitioned into indirect (cancer risk associated with IGF-I/IR traits–related SNPs mediated by IGF-I/IR traits) and direct effects (cancer risk associated with IGF-I/IR traits–related SNPs via pathways other than IGF-I/IR traits). This approach allowed us to test the hypothesis that IGF-I/IR traits–related SNPs are associated with risk of CRC and that the relationships depend on IGF-I/IR traits. A total and direct effect size of IGF-I/IR traits–related SNPs (exposure) on CRC (outcome) was produced from the HR for IGF-I/IR traits–related SNPs predicting CRC in the Cox model that included all covariates, without (total) and with (direct) IGF-I/IR traits (mediator). The indirect effect size was produced via a traditional statistical approach [52]: the percentage change in the HRs by comparing a model that includes all covariates with a model that includes all covariates and the mediator.

**Fig 2:**
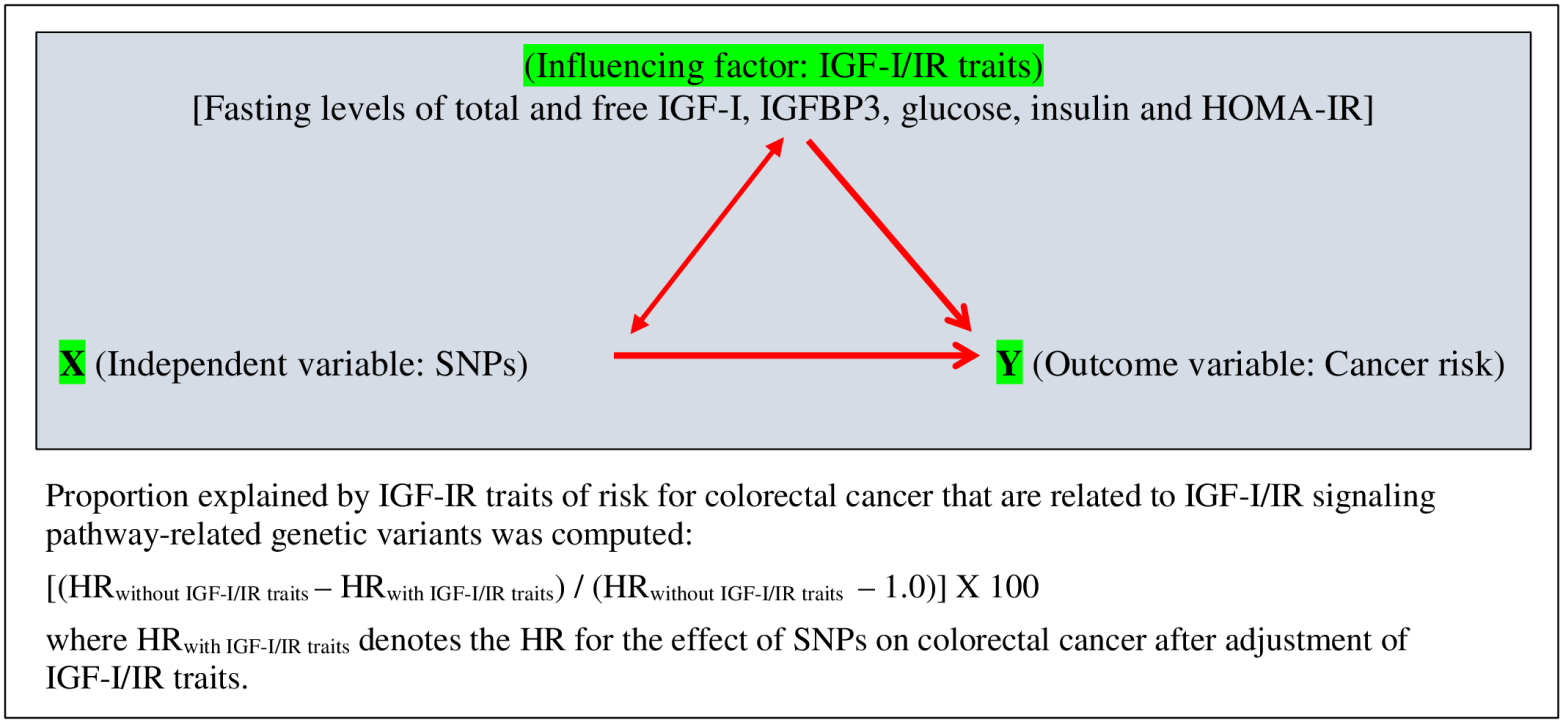
Pathways of SNPs in IGF-I/IR *signaling pathways*–related genes, IGF-I/IR traits, and colorectal cancer risk. (HOMA-IR, homeostatic model assessment–insulin resistance; HR, hazard ratio; IGF-I, insulin-like growth factor-I; IGFBP3, IGF binding protein 3; IR, insulin resistance; SNP, single-nucleotide polymorphism) Proportion explained by IGF-IR traits of risk for colorectal cancer that are related to IGF-I/IR signaling pathway-related genetic variants was computed: [(HRwithout IGF-I/IR traits−HR_with IGF-I/IR traits_) / (HR_without IGF-I/IR traits_− 1.0)] X 100 where HR_with IGF-I/IR traits_ denotes the HR for the effect of SNPs on colorectal cancer after adjustment of IGF-I/IR traits.

Next, IGF-I/IR traits are not mediators in the relationship between IGF-I/IR signaling pathways–relevant SNPs and CRC; we examined the effect of IGF-I/IR traits as an influencing factor on these SNPs–cancer associations. (Fig 2). The effect of IGF-I/IR traits on CRC risk that is associated with IGF-I/IR signaling pathways–relevant SNPs was estimated using the same algorithm as that of mediator, but it was interpreted as an influential factor.

To evaluate the role of obesity, PA, and exogenous E as effect modifiers on the pathway of IGF-I/IR SNPs, IGF-I/IR traits, and CRC, we stratified participants by those potential effect modifiers and within the strata, compared the proportions of the cancer risk contributed by IGF-I/IR SNPs through IGF-I/IR traits (indirect effect) and non–IGF-I/IR traits pathways (direct effect). A 2-tailed p value < 0.05 was considered statistically significant. The R statistical package (v 2.15.1) was used.

## Results

Participants’ baseline characteristics and allele frequencies of 33 SNPs by obese status, level of PA, exogenous E use, and CRC status are presented in S1–S13 Tables. The participants had been followed up through February 29, 2004, resulting in 237 cases of CRC (32% of non-obese vs. 40% of obese women; 31% of active vs. 36% of inactive women; and 40% of nonusers vs. 31% of E-only users vs. 29% of E+P users are CRC cases).

### CRC risk associated with IGF-I/IR *trait*–related SNPs that is mediated via IGF-I/IR traits, stratified by obesity status (BMI, waist, and w/h), level of PA, and exogenous E use

We partitioned the total effect of IGF-IR/IR trait–related SNPs on CRC risk into direct (not via IGF-I/IR traits) and indirect (via IGF-I/IR traits) effects (Fig 1). Each SNP associated with CRC in this analysis was mediated via fasting levels of total and free IGF-I, IGFBP3, insulin, glucose, and HOMA-IR. For each SNP with mediators, the IGF-I/IR trait SNP–CRC association was evaluated by stratification via obesity status (BMI < 30 kg/m^2^ vs. ≥ 30 kg/m^2^; waist ≤ 88 cm vs. > 88 cm; and w/h ≤ 0.85 vs. > 0.85), level of PA (metabolic equivalent [MET hours·week^-1^] ≥ 10 vs. < 10) (Table 1), and by exogenous E use (nonuse vs. E-only or E+P use) (Table 2).

**Table 1:** Mediation (indirect) effect of Metabolic Biomarker Levels on the relationship between IGFs/IR–relevant SNPs and CRC risk, stratified by obesity status‡ and physical activity level.

SNP_¥	Effect allele/Other allele	Non-Obese or Active Group							Obese or Inactive Group						
		Total effect£			Direct effect£			Indirect effect*	Total effect£			Direct effect£			Indirect effect*
		CRC risk in relation to SNP			CRC risk in relation to SNP through pathways other than *biomarker*			CRC risk in relation to SNP through *biomarker*	CRC risk in relation to SNP			CRC risk in relation to SNP through pathways other than *biomarker*			CRC risk in relation to SNP through *biomarker*
		HR	95% CI		HR	95% CI		%	HR	95% CI		HR	95% CI		%
		**A. Obesity status**													
		**BMI < 30 kg/m**^**2**^ **(n = 515)**							**BMI ≥ 30 kg/m**^**2**^ **(n = 174)**						
								***Insulin level***							***Insulin level***
***INS*RS689_AD**	**T/A**	1.26	0.94	1.68	**1.36**	**1.01**	**1.83**	38.40	0.70	0.40	1.22	0.69	0.39	1.22	1.92
***INS*RS689_D**	**T/A**	1.37	0.96	1.98	**1.49**	**1.02**	**2.17**	30.34	0.62	0.30	1.27	0.57	0.27	1.21	7.30
								***HOMA-IR level***							***HOMA-IR level***
***INS*RS689_AD**	**T/A**	1.26	0.94	1.68	**1.36**	**1.01**	**1.84**	40.17	0.71	0.41	1.24	0.67	0.38	1.19	5.54
***INS*RS689_D**	**T/A**	1.37	0.96	1.98	**1.49**	**1.02**	**2.18**	31.31	0.64	0.31	1.31	0.58	0.27	1.22	9.93
		**B. Physical activity level**													
		**Active Group (MET ≥ 10, n = 348)**							**Inactive Group (MET < 10, n = 356)**						
								***Total IGF-I level***							***Total IGF-I level***
***IGF-I*RS10860865_D**	**T/G**	**0.60**	**0.37**	**0.97**	0.62	0.39	1.01	3.67	1.21	0.78	1.89	1.14	0.73	1.80	32.15
***IGF-I*RS10860865_R**	**T/G**	1.39	0.57	3.34	1.26	0.52	3.05	32.23	**2.97**	**1.25**	**7.04**	2.45	1.00	5.96	26.53

BMI, body mass index; CRC, colorectal cancer; CI, confidence interval; HOMA-IR, homeostatic model assessment–insulin resistance; HR, hazard ratio; IGF-I, insulin-like growth factor-I; IR, insulin resistance; MET, metabolic equivalent; SNP, single-nucleotide polymorphism.

Note: Among SNPs having statistically significant association with cancer in either subgroup, the SNPs with ≥ 30% of indirect effect in this subgroup or its counterpart are included. Numbers in bold face are statistically significant.

^‡^ Lifestyle modifiers whose strata include statistically significant association between SNP and cancer only are presented.

^¥^ Tag attached to the SNP name indicates the SNP-analysis approach (AD: additive; D: minor-allele dominant; and R: minor-allele recessive).

^£^ Multivariate regression was adjusted by covariates (age, education, family income, family histories of diabetes mellitus or colorectal cancer, heart failure ever, high cholesterol requiring pills ever, smoking status, alcohol intake, and reproductive history [oral contraceptive and history of hysterectomy or oophorectomy, ages at menarche and menopause, and history of pregnancy]); effect-modifier variables (physical activity, BMI, and exogenous estrogen use), when not evaluated as effect modifier variables, were adjusted as a covariate; when stratified via waist circumference or waist-to-hip ratio, BMI was not adjusted.

* Indirect effect estimated via the proportional difference between the HRs without (total effect) and with (direct effect) accounting for hormone.

**Table 2:** Mediation (Indirect) effect of Metabolic Biomarker Levels on the relationship between IGFs/IR–relevant SNPs and CRC risk, stratified by exogenous estrogen use status.

SNP_¥	Effect allele/Other allele	Nonuser Group							User Group						
		Total effect£			Direct effect£			Indirect effect*	Total effect£			Direct effect£			Indirect effect*
		CRC risk in relation to SNP			CRC risk in relation to SNP through pathways other than *biomarker*			CRC risk in relation to SNP through *biomarker*	CRC risk in relation to SNP			CRC risk in relation to SNP through pathways other than *biomarker*			CRC risk in relation to SNP through *biomarker*
		HR	95% CI		HR	95% CI		%¶	HR	95% CI		HR	95% CI		%¶
		**Nonusers (n = 269)**							**E only users (n = 199)**						
								***Insulin level***							***Insulin level***
***INS*RS689_R**	**T/A**	2.24	0.74	6.76	**3.34**	**1.07**	**10.47**	89.20	0.87	0.27	2.80	0.73	0.18	2.99	15.81
		**Nonusers (n = 273)**							**E+P users (n = 178)**						
								***Total IGF-I level***							***Total IGF-I level***
***IGF-I*RS10778176_R**	**T/C**	2.61	1.02	6.69	**2.70**	**1.03**	**7.05**	5.31	2.35	0.76	7.25	2.78	0.89	8.72	32.17
								***IGFBP3 level***							***IGFBP3 level***
***IGFBP3*RS2471551_AD**	**C/G**	**0.53**	**0.32**	**0.86**	**0.54**	**0.33**	**0.89**	2.93	1.21	0.68	2.16	1.35	0.74	2.48	64.96
***IGFBP3*RS2471551_D**	**C/G**	**0.39**	**0.22**	**0.69**	**0.40**	**0.23**	**0.71**	2.80	1.51	0.69	3.31	1.75	0.76	4.05	49.05
***IGFBP3*RS3110697_D**	**A/G**	**0.50**	**0.28**	**0.88**	**0.53**	**0.30**	**0.97**	7.70	1.62	0.69	3.78	1.87	0.74	4.73	40.23
								***Insulin level***							***Insulin level***
***INS*RS3842763_D**	**A/C**	0.58	0.34	0.99	**0.52**	**0.30**	**0.90**	9.67	1.35	0.63	2.90	1.22	0.55	2.69	37.36
***INS*RS689_R**	**T/A**	2.24	0.74	6.76	**3.34**	**1.07**	**10.47**	89.20	1.42	0.45	4.43	1.54	0.48	5.00	29.32
								***HOMA-IR level***							***HOMA-IR level***
***INS*RS689_R**	**T/A**	2.24	0.74	6.76	**3.55**	**1.13**	**11.13**	N/A	1.41	0.45	4.38	1.21	0.34	4.26	48.68

CI, confidence interval; CRC, colorectal cancer; E, exogenous estrogen; HOMA-IR, homeostatic model assessment–insulin resistance; HR, hazard ratio; IGF-I, insulin-like growth factor-I; IGFBP3, IGF binding protein 3; IR, insulin resistance; P, progestin; SNP, single-nucleotide polymorphism.

Note: Among SNPs having statistically significant association with cancer in either subgroup, the SNPs with ≥ 30% of indirect effect in this subgroup or its counterpart are included. Numbers in bold face are statistically significant.

^¥^ Tag attached to the SNP name indicates the SNP-analysis approach (AD: additive; D: minor-allele dominant; and R: minor-allele recessive).

^£^ Multivariate regression was adjusted by covariates (age, education, family income, family histories of diabetes mellitus or colorectal cancer, heart failure ever, high cholesterol requiring pills ever, smoking status, alcohol intake, and reproductive history [oral contraceptive and history of hysterectomy or oophorectomy, ages at menarche and menopause, and history of pregnancy]); effect-modifier variables (physical activity, BMI, and exogenous estrogen use), when not evaluated as effect modifier variables, were adjusted as a covariate; when stratified via waist circumference or waist-to-hip ratio, body mass index was not adjusted.

* Indirect effect estimated via the proportional difference between the HRs without (total effect) and with (direct effect) accounting for hormone.

^¶^ Not applicable due to either ≥ 50% difference between small effect sizes or ≥ 100% difference between two effect sizes.

Of 19 IGF-I/IR trait–related SNPs, a few SNPs in the *INS*, *IGF-I*, and *IGFBP3* genes were significantly associated with CRC risk (Tables 1 and 2). Overall, the SNP–cancer association differed between the strata (non-obese/active vs. obese/inactive; E nonuse vs. use). In all strata, the direct effect (not via IGF-I/IR traits) of the SNP–cancer risk was dominant in each SNP over the indirect effect (via IGF-I/IR traits) regardless of the mediator.

Carriers of the *INS* rs689 T allele had an increased CRC risk among the non-obese (BMI < 30) group (Table 1). Roughly 35% of the CRC risk owing to this SNP was mediated via insulin or HOMA-IR levels in this group. However, a different mediation effect of this trait was observed when stratified by exogenous E use status. In the nonuser group, where increased CRC risk was found, the mediation effect of insulin level on this SNP–cancer association was strong (> 80%) (Table 2). When participants were classified by exogenous E use status, 4 SNPs in nonusers were associated with CRC risk (Table 2): the *IGF-I* rs10778176 T allele, with increased risk, and the *IGFBP3* rs2471551 C and rs3110697 A alleles and the *INS* rs3842763 A allele, with decreased risk. The mediation effect of the relevant trait in this nonuse group was minimal.

### CRC risk associated with IGF-I/IR *signaling pathway*–related SNPs and IGF-I/IR traits, stratified by obesity status (BMI, waist, and w/h), level of PA, and exogenous E use

Because IGF-I/IR traits are not mediators of the association between SNPs in IGF-I/IR signaling-pathway genes (*IRS1* and *AKT1/2*, in this study) and CRC, instead of estimating the mediation effect of the traits, we estimated a proportion explained by the traits as an influencing factor of the SNP–cancer relationship (Fig 2). For each SNP, the proportion was estimated using the traits (fasting levels of total and free IGF-I, IGFBP3, insulin, glucose, and HOMA-IR), after stratification by obesity status, level of PA, and exogenous E use (Tables 3–5). Of 14 SNPs in the IGF-I/IR signaling pathway–related genes, more than two thirds were associated significantly with CRC risk. Overall, the SNP–cancer association differed by obesity, PA, and exogenous E use. In addition, the proportion explained by the traits of the SNP–cancer association differed between the strata.

**Table 3:** Effect of Metabolic Biomarker Levels on the relationship between IGF-I/IR signaling pathways–relevant SNPs (in *IRS1* gene) and CRC risk, stratified by selected lifestyle factors‡.

SNP_¥	Effect allele/Other allele	Active or Nonuser Group							Inactive or User Group						
		CRC risk in relation to SNP			CRC risk in relation to SNP adjusted by *biomarker*			Proportion of relationship between CRC and SNP explained by *biomarker* *	CRC risk in relation to SNP			CRC risk in relation to SNP adjusted by *biomarker*			Proportion of relationship between CRC and SNP explained by *biomarker* *
		HR£	95% CI		HR£	95% CI		%	HR£	95% CI		HR£	95% CI		%
		**A. Physical activity level**													
		**Active Group (MET ≥ 10, n = 348)**							**Inactive Group (MET < 10, n = 356)**						
								***Total IGF-I level***							***Total IGF-I level***
***IRS1*RS1801123_D**	**G/A**	0.92	0.51	1.65	1.01	0.56	1.82	9.43	1.58	0.91	2.74	**1.98**	**1.11**	**3.53**	69.49
								***Insulin level***							***Insulin level***
***IRS1*RS1801278_AD**	**T/C**	1.28	0.65	2.49	1.27	0.64	2.51	3.85	1.63	0.84	3.19	**2.20**	**1.10**	**4.38**	89.01
***IRS1*RS1801278_D**	**T/C**	1.10	0.53	2.28	1.08	0.51	2.28	16.37	1.85	0.90	3.79	**2.54**	**1.21**	**5.31**	82.25
								***HOMA-IR level***							***HOMA-IR level***
***IRS1*RS1801278_D**	**T/C**	1.12	0.54	2.32	1.10	0.52	2.32	12.23	1.85	0.90	3.79	**2.20**	**1.06**	**4.56**	41.58
		**B. Exogenous estrogen use**													
		**Nonusers (n = 273)**							**E only users (n = 205)**						
								***Total IGF-I level***							***Total IGF-I level***
***IRS1*RS1801278_AD**	**T/C**	1.25	0.60	2.59	1.32	0.62	2.84	32.07	**2.71**	**1.05**	**6.97**	**3.10**	**1.15**	**8.36**	22.72
								***Free IGF-I level***							***Free IGF-I level***
***IRS1*RS1801278_AD**	**T/C**	1.12	0.53	2.36	1.08	0.51	2.30	30.83	**2.81**	**1.11**	**7.10**	**2.80**	**1.11**	**7.10**	0.47
								***Insulin level***							***Insulin level***
***IRS1*RS1801278_D**	**T/C**	1.36	0.61	3.01	1.47	0.66	3.29	32.03	**3.48**	**1.08**	**11.26**	4.87	0.72	32.98	55.58
								***Glucose level***							***Glucose level***
***IRS1*RS1801278_D**	**T/C**	1.38	0.62	3.06	1.57	0.70	3.55	50.51	**3.58**	**1.10**	**11.60**	**3.55**	**1.09**	**11.55**	1.10
								***HOMA-IR level***							***HOMA-IR level***
***IRS1*RS1801278_AD**	**T/C**	1.23	0.59	2.57	1.37	0.65	2.90	59.91	2.59	0.99	6.76	**3.05**	**1.03**	**9.07**	28.96
***IRS1*RS1801278_D**	**T/C**	1.36	0.61	3.01	1.50	0.67	3.37	39.59	**3.48**	**1.08**	**11.26**	**4.40**	**1.10**	**17.62**	36.98

CI, confidence interval; CRC, colorectal cancer; HOMA-IR, homeostatic model assessment–insulin resistance; HR, hazard ratio; IGF-I, insulin-like growth factor-I; IR, insulin resistance; MET, metabolic equivalent; SNP, single–nucleotide polymorphism. Note: Among SNPs having statistically significant association with cancer in either subgroup, the SNPs with ≥ 30% of indirect effect in this subgroup or its counterpart are included. Numbers in bold face are statistically significant.

^‡^ Lifestyle modifiers whose strata include statistically significant association between SNP and cancer only are presented.

^¥^ Tag attached to the SNP name indicates the SNP-analysis approach (AD: additive; D: minor-allele dominant; and R: minor-allele recessive).

^£^ Multivariate regression was adjusted by covariates (age, education, family income, family histories of diabetes mellitus or colorectal cancer, heart failure ever, high cholesterol requiring pills ever, smoking status, alcohol intake, and reproductive history [oral contraceptive and history of hysterectomy or oophorectomy, ages at menarche and menopause, and history of pregnancy]); effect-modifier variables (physical activity, BMI, and exogenous estrogen use), when not evaluated as effect modifier variables, were adjusted as a covariate; when stratified via waist circumference or waist-to-hip ratio, body mass index was not adjusted.

* Proportional difference was estimated via difference in the HRs without (total effect) and with (direct effect) accounting for hormone.

**Table 4:** Effect of Metabolic Biomarker Levels on the relationship between IGF-I/IR signaling pathways–relevant SNPs (in *AKT1/2* genes) and CRC risk, stratified by obesity status‡.

SNP_¥	Effect allele/Other allele	Non-Obese Group							Obese Group						
		CRC risk in relation to SNP			CRC risk in relation to SNP adjusted by *biomarker*			Proportion of relationship between CRC and SNP explained by *biomarker* *	CRC risk in relation to SNP			CRC risk in relation to SNP adjusted by *biomarker*			Proportion of relationship between CRC and SNP explained by *biomarker* *
		HR£	95% CI		HR£	95% CI		%	HR£	95% CI		HR£	95% CI		%
		**BMI < 30 kg/m**^**2**^ **(n = 525)**							**BMI ≥ 30 kg/m**^**2**^ **(n = 177)**						
								***IGFBP3 level***							***IGFBP3 level***
***AKT2*RS2304186_AD**	**A/C**	1.22	0.94	1.58	1.22	0.94	1.58	0.37	1.64	0.97	2.75	**1.83**	**1.05**	**3.19**	29.70
***AKT2*RS2304186_R**	**A/C**	1.26	0.79	1.99	1.26	0.79	1.99	0.27	2.33	0.93	5.84	**3.15**	**1.13**	**8.78**	61.23
								***HOMA-IR level***							***HOMA-IR level***
***AKT2*RS3730256_D**	**T/C**	**0.55**	**0.33**	**0.92**	**0.57**	**0.34**	**0.96**	3.20	0.76	0.27	2.11	0.53	0.17	1.66	30.47
		**Waist ≤ 88 cm (n = 445)**							**Waist > 88 cm (n = 244)**						
								***Insulin level***							***Insulin level***
***AKT2*RS11673367_R**	**A/T**	**0.28**	**0.08**	**0.96**	**0.23**	**0.07**	**0.81**	18.11	1.60	0.51	4.99	1.06	0.27	4.14	90.50
								***Glucose level***							***Glucose level***
***AKT2*RS11673367_R**	**A/T**	**0.28**	**0.08**	**0.93**	**0.27**	**0.08**	**0.90**	2.65	1.54	0.50	4.76	1.88	0.59	5.99	62.35
								***HOMA-IR level***							***HOMA-IR level***
***AKT2*RS2304186_AD**	**A/C**	1.35	1.00	1.82	**1.38**	**1.03**	**1.86**	8.20	1.09	0.74	1.60	1.13	0.76	1.68	45.53
		**w/h Ratio ≤ 0.85 (n = 533)**							**w/h Ratio > 0.85 (n = 171)**						
								***Total IGF-I level***							***Total IGF-I level***
***AKT2*RS7247515_AD**	**A/G**	**0.49**	**0.27**	**0.90**	**0.49**	**0.27**	**0.90**	0.41	1.12	0.39	3.27	1.17	0.40	3.40	37.11
								***Free IGF-I level***							***Free IGF-I level***
***AKT2*RS11673367_AD**	**A/T**	**0.63**	**0.45**	**0.87**	**0.63**	**0.45**	**0.87**	0.13	1.30	0.73	2.33	1.19	0.65	2.17	36.97
***AKT2*RS11673367_D**	**A/T**	**0.65**	**0.44**	**0.96**	**0.65**	**0.44**	**0.96**	0.18	1.11	0.55	2.26	1.01	0.49	2.08	94.39
								***IGFBP3 level***							***IGFBP3 level***
***AKT2*RS11673367_D**	**A/T**	**0.65**	**0.44**	**0.96**	**0.65**	**0.44**	**0.96**	0.25	1.13	0.57	2.26	1.19	0.59	2.39	42.34
								***Insulin level***							***Insulin level***
***AKT1*RS1130214_AD**	**T/G**	**0.73**	**0.54**	**0.99**	0.73	0.54	1.00	0.96	1.11	0.65	1.90	1.18	0.51	2.71	59.30
***AKT1*RS1130214_D**	**T/G**	**0.60**	**0.41**	**0.88**	**0.60**	**0.41**	**0.89**	0.68	1.19	0.59	2.40	1.04	0.42	2.56	81.57
***AKT2*RS11673367_AD**	**A/T**	**0.68**	**0.49**	**0.94**	**0.66**	**0.47**	**0.91**	3.28	1.27	0.71	2.27	1.10	0.49	2.45	62.08
***AKT2*RS11673367_R**	**A/T**	**0.22**	**0.06**	**0.73**	**0.17**	**0.05**	**0.61**	21.13	3.37	0.80	14.08	1.98	0.20	19.15	58.72
***AKT2*RS7247515_D**	**A/G**	**0.48**	**0.26**	**0.90**	**0.46**	**0.25**	**0.87**	4.06	0.87	0.28	2.73	0.55	0.09	3.42	36.17
								***HOMA-IR level***							***HOMA-IR level***
***AKT1*RS1130214_D**	**T/G**	**0.59**	**0.40**	**0.87**	**0.60**	**0.40**	**0.88**	1.05	1.19	0.59	2.40	1.26	0.53	2.99	34.36
***AKT2*RS11673367_AD**	**A/T**	**0.69**	**0.50**	**0.95**	**0.66**	**0.48**	**0.92**	3.34	1.27	0.71	2.27	1.15	0.58	2.26	45.06
***AKT2*RS11673367_R**	**A/T**	**0.22**	**0.07**	**0.73**	**0.17**	**0.05**	**0.60**	24.51	3.37	0.80	14.08	2.51	0.46	13.83	35.99
***AKT2*RS3730256_D**	**T/C**	**0.41**	**0.24**	**0.72**	**0.40**	**0.23**	**0.70**	3.27	1.23	0.46	3.29	1.09	0.32	3.72	59.15

BMI, body mass index; CI, confidence interval; CRC, colorectal cancer; HOMA-IR, homeostatic model assessment–insulin resistance; HR, hazard ratio; IGF-I, insulin-like growth factor-I; IGFBP3, IGF binding protein 3; IR, insulin resistance; SNP, single-nucleotide polymorphism; w/h ratio, waist-to-hip ratio. Note: Among SNPs having statistically significant association with cancer in either subgroup, the SNPs with ≥ 30% of indirect effect in this subgroup or its counterpart are included. Numbers in bold face are statistically significant.

^‡^ Lifestyle modifiers whose strata include statistically significant association between SNP and cancer only are presented.

^¥^ Tag attached to the SNP name indicates the SNP-analysis approach (AD: additive; D: minor-allele dominant; and R: minor-allele recessive).

^£^ Multivariate regression was adjusted by covariates (age, education, family income, family histories of diabetes mellitus or colorectal cancer, heart failure ever, high cholesterol requiring pills ever, smoking status, alcohol intake, and reproductive history [oral contraceptive and history of hysterectomy or oophorectomy, ages at menarche and menopause, and history of pregnancy]); effect-modifier variables (physical activity, BMI, and exogenous estrogen use), when not evaluated as effect modifier variables, were adjusted as a covariate; when stratified via waist circumference or waist-to-hip ratio, BMI was not adjusted.

* Proportional difference was estimated via difference in the HRs without (total effect) and with (direct effect) accounting for hormone.

**Table 5:** Effect of Metabolic Biomarker Levels on the relationship between IGF-I/IR signaling pathways–relevant SNPs (in *AKT1/2* genes) and CRC risk, stratified by physical activity level and exogenous estrogen use status ‡.

SNP_¥	Effect allele/Other allele	Active, or Nonuser Group							Inactive, or User Group						
		CRC risk in relation to SNP			CRC risk in relation to SNP adjusted by *biomarker*			Proportion of relationship between CRC and SNP explained by *biomarker* *	CRC risk in relation to SNP			CRC risk in relation to SNP adjusted by *biomarker*			Proportion of relationship between CRC and SNP explained by *biomarker* *
		HR£	95% CI		HR£	95% CI		%¶	HR£	95% CI		HR£	95% CI		%¶
		**A. Physical activity level**													
		**Active Group (MET ≥ 10, n = 344)**							**Inactive Group (MET < 10, n = 345)**						
								***Insulin level***							***Insulin level***
***AKT2*RS4332845_D**	**A/T**	**0.52**	**0.32**	**0.85**	**0.54**	**0.33**	**0.89**	3.65	1.27	0.81	2.01	1.39	0.87	2.23	44.25
								***HOMA-IR level***							***HOMA-IR level***
***AKT2*RS11673367_R**	**A/T**	0.37	0.10	1.40	0.38	0.10	1.42	1.64	0.56	0.21	1.49	**0.30**	**0.09**	**0.94**	47.13
***AKT2*RS4332845_D**	**A/T**	**0.53**	**0.33**	**0.87**	**0.55**	**0.34**	**0.91**	3.61	1.27	0.81	2.01	1.42	0.89	2.27	55.79
		**B. Exogenous estrogen status**													
		**Nonusers (n = 269)**							**E+P users (n = 176)**						
								***Insulin level***							***Insulin level***
***AKT1*RS2494738_AD**	**T/C**	**2.56**	**1.16**	**5.66**	**2.81**	**1.25**	**6.32**	15.83	**0.11**	**0.02**	**0.55**	**0.08**	**0.01**	**0.44**	30.87
***AKT1*RS2494738_D**	**T/C**	2.19	0.98	4.89	**2.39**	**1.05**	**5.45**	17.62	**0.11**	**0.02**	**0.53**	**0.08**	**0.01**	**0.43**	30.33
***AKT1*RS2494740_D**	**T/A**	1.01	0.60	1.71	1.00	0.59	1.70	N/A	**2.51**	**1.13**	**5.60**	**3.24**	**1.25**	**8.40**	47.73
***AKT1*RS2498789_AD**	**C/T**	**1.94**	**1.07**	**3.52**	**1.99**	**1.09**	**3.64**	5.05	0.51	0.18	1.49	0.31	0.08	1.13	40.09

CI, confidence interval; CRC, colorectal cancer; HOMA-IR, homeostatic model assessment–insulin resistance; HR, hazard ratio; IGF-I, insulin-like growth factor-I; IR, insulin resistance; MET, metabolic equivalent; SNP, single-nucleotide polymorphism. Note: Among SNPs having statistically significant association with cancer in either subgroup, the SNPs with ≥ 30% of indirect effect in this subgroup or its counterpart are included. Numbers in bold face are statistically significant.

^‡^ Lifestyle modifiers whose strata include statistically significant association between SNP and cancer only are presented.

^¥^ Tag attached to the SNP name indicates the SNP-analysis approach (AD: additive; D: minor-allele dominant; and R: minor-allele recessive).

^£^ Multivariate regression was adjusted by covariates (age, education, family income, family histories of diabetes mellitus or colorectal cancer, heart failure ever, high cholesterol requiring pills ever, smoking status, alcohol intake, and reproductive history [oral contraceptive and history of hysterectomy or oophorectomy, ages at menarche and menopause, and history of pregnancy]); effect-modifier variables (physical activity, BMI, and exogenous estrogen use), when not evaluated as effect modifier variables, were adjusted as a covariate; when stratified via waist circumference or waist-to-hip ratio, BMI was not adjusted.

* Proportional difference was estimated via difference in the HRs without (total effect) and with (direct effect) accounting for hormone.

^¶^ Not applicable due to either ≥ 50% difference between small effect sizes or ≥ 100% difference between two effect sizes.

In relation to the SNPs in the *IRS1* gene (Table 3), carriers of the *IRS1* rs1801123 G and rs1801278 T alleles had increased CRC risk in inactive (MET < 10) women, with roughly 50% of CRC risk due to each SNP that was explained by traits. Further, carriers of the *IRS1* rs1801278 T allele, when stratified by exogenous E use, had increased risk of CRC in E-only users; approximately 30% of the CRC risk associated with this SNP was dependent on traits.

Several SNPs in the *AKT1* and *AKT2* genes were significantly associated with CRC risk (Tables 4 and 5). When stratified by obesity status and PA level, carriers of the following SNPs had decreased risk of CRC in non-obese women: *AKT2* rs11673367 A allele (in waist ≤ 88, w/h ≤ 0.85, and MET ≥ 10 groups); *AKT2* rs3730256 T allele (in BMI < 30 and w/h ≤ 0.85 groups); *AKT2* rs7247515 A allele (in w/h ≤ 0.85 group); *AKT2* rs4332845 A allele (in MET ≥ 10 group); and *AKT1* rs1130214 T allele (in w/h ≤ 0.85 group). The effects of the traits on the SNP–cancer association in those groups were negligible. Differently, carriers of one SNP (rs2304186 A allele) in the *AKT2* gene in the obese (BMI ≥ 30) group had increased risk of CRC, and about 50% of the CRC risk due to this SNP was explained by IGFBP3 levels (Tables 4 and 5).

Further, when stratified by exogenous E use, carriers of the *AKT1* rs2494738 T and rs2498789 C alleles had an increased risk of CRC among nonusers; however, in E+P users, carriers of the rs2494738 T allele had a reduced risk of CRC (with 30% of the SNP–cancer association explained by insulin level). In contrast, carriers of the *AKT1* rs2494740 T allele had an increased risk of CRC among E+P users, with about 50% of this SNP–cancer association explained by insulin level (Table 5).

## Discussion

This study, to our knowledge, is the first to evaluate in postmenopausal women the association between IGF-I/IR-related genetic variants and CRC risk using mediation analysis to determine the extent to which CRC–SNP relationship is explained by metabolic biomarkers (i.e., IGF/IR traits). Additionally, we examined whether lifestyle factors, such as obesity, PA, and exogenous E use, modified the pathway connecting the genetic variant, trait, and CRC risk. Our major finding was that there are a number of significant associations between the IGF/IR-axis SNPs studied and CRC risk, many of which are mediated by circulating levels of metabolic biomarkers. However, these associations would be missed unless the analyses are stratified by obesity, PA level, and use of exogenous E.

Among the 33 IGF/IR-related SNPs we evaluated, 6 (of the 19 IGF-IR traits) in the *INS*, *IGF-I*, and *IGFBP3* genes and 11 (of the 14 IGF/IR signaling pathway) in the *IRS1* and *AKT1/2* genes were associated with CRC risk. The association of these SNPs with CRC risk differed between strata (non-obese/active vs. obese/inactive women; E nonusers vs. users), indicating that lifestyle factors (obesity, PA, and exogenous E) modified the SNP–cancer association. For most of those SNPs that were associated with CRC in this study, the direct effect on cancer risk accounted for a majority of the total effect; roughly 30% of CRC risk associated with the SNPs was explained via IGF-I/IR traits. This suggests that the traits are not the main mediators through which IGF-I/IR SNPs are associated with CRC risk, warranting further study of the pathway (e.g., dietary or inflammatory pathways).

In our study, carriers of the *INS* rs689 T allele had increased CRC risk in non-obese and E nonusers, with 35% and 80%, respectively, of the SNP–cancer association mediated by insulin/HOMA-IR levels. Two previous studies [13, 53] examined the association between the *INS* rs689 and CRC risk, finding no significant association with CRC. Our study is the first to show a significant association of this SNP with CRC risk, but only among non-obese and E nonusers, with modest and strong mediation effects of traits. These findings suggest that the carcinogenesis pathway in this SNP interacts with the glucose-intolerance system, and further study is needed to evaluate the implication of obesity and estrogen in this tumorigenesis mechanism.

The insulin receptor substrate-1 (IRS-1) is a main substrate initiating and directing IGF-I/1R signaling. Genetic alteration in the relevant gene is associated with impaired downstream signaling, leading to insulin resistance and probably cancer.[22] Several previous studies evaluated the association between the *IRS1* rs1801278 (Gly972Arg) and CRC risk and showed inconsistent findings: no significant relationship [13, 18], increased risk [11, 17], and decreased risk [10] of CRC with the T allele (vs. the C allele) in this SNP were examined. In our study, those carriers had an increased risk of CRC among inactive and E only users, suggesting that an obesity-related lifestyle and exogenous estrogen play a role in modulating the effect of this SNP on carcinogenesis.

Of 3 members (AKt1, AKt2, and AKt3) of the AKt family, AKt1 and AKt2 are important signaling molecules related to a diabetic phenotype such as IR; at the genomic level, each is amplified in various cancers including breast cancer [54, 55]. The *AKT1/2* genes are thus key components of this pathway, but studies of the association of their genetic variants with CRC have been limited. Consistent with one previous study [56] showing that genetic variants in the insulin pathway were associated with CRC by interacting with lifestyle factors (e.g., diet), we found that several SNPs in the *AKT1/2* genes were associated with CRC, by interacting with obesity, PA, and exogenous E use. Carriers of several SNPs in this AKt pathway were associated with increased risk of CRC in obese/inactive women and decreased risk in non-obese/active women; this indicates that the signaling pathway–related carcinogenesis in these SNPs communicates with adiposity.

An interesting note was that when stratified by exogenous E use status, some SNPs in the same molecular pathway had different associations with CRC among E users. For example, in E+P users, carriers of 2 SNPs in the *AKT1* gene had discrepant associations with CRC: the rs2494749 T allele was associated with increased risk, and the rs2494738 T allele, with decreased risk. This suggests that estrogen’s cross talk with the target gene downstream of the signaling pathway affects cancer risk [24–28] and that the extent of this interaction may be SNP-specific and dependent on the combination of hormone therapy, but the clear mechanism is unknown.

We did not include any multiple-testing adjustments in our analyses. We tested the hypothesis that the interactions between genetic variants and lifestyle factors influence IGFs/IR traits, resulting in altered cancer risk. We acknowledge that, as with many analyses, we might have a few false-positive results and that the results should be interpreted with care, especially when p values are close to the level of significance. Also, our findings from the mediation and proportion approach should only be interpreted statistically and do not necessarily imply any functional connections. Some analyses after stratification had large CIs with null associations due to small sample sizes. Finally, our study analyzed the data from non–Hispanic white postmenopausal women only, so, the generalizability of our findings to other populations is limited. Despite these limitations, the potential impact of our findings clearly warrants further study.

In conclusion, our findings suggest that in postmenopausal women, the IGF-I/IR axis has a potential role in the risk for CRC. Lifestyle factors including obesity, PA, and exogenous E use modulate the association between IGF-I/IR genetic variants and cancer, partially through IGF-I/IR traits. Further studies are needed to explore these complex mechanisms. Our results provide insight into gene–lifestyle interactions and suggest data on potential genetic targets for use in clinical trials for cancer prevention and intervention strategies to reduce the risk for CRC in postmenopausal women.

## Supporting information

S1 Fig Flow diagram of analytic cohort. (DOCX)

S1 Table Allele frequencies of 33 IGF-I/insulin pathways–relevant SNPs, stratified by obesity (measured via BMI). (DOCX)

S2 Table Allele frequencies of 33 IGF-I/insulin pathways–relevant SNPs, stratified by obesity (measured via waist circumference). (DOCX)

S3 Table Allele frequencies of 33 IGF-I/insulin pathways–relevant SNPs, stratified by obesity (measured via w/h ratio). (DOCX)

S4 Table Allele frequencies of 33 IGF-I/insulin pathways–relevant SNPs, stratified by physical activity level. (DOCX)

S5 Table Allele frequencies of 33 IGF-I/insulin pathways–relevant SNPs, stratified by exogenous estrogen use (nonusers vs. E-only users). (DOCX)

S6 Table Allele frequencies of 33 IGF-I/insulin pathways–relevant SNPs, stratified by exogenous estrogen use (nonusers vs. E+P users). (DOCX)

S7 Table Characteristics of participants, stratified by obesity (measured via BMI). (DOCX)

S8 Table Characteristics of participants, stratified by obesity (measured via waist circumference). (DOCX)

S9 Table Characteristics of participants, stratified by obesity (measured via w/h ratio). (DOCX)

S10 Table Characteristics of participants, stratified by physical activity level. (DOCX)

S11 Table Characteristics of participants, stratified by exogenous estrogen use (nonusers vs. E-only users). (DOCX)

S12 Table Characteristics of participants, stratified by exogenous estrogen use (nonusers vs. E+P users). (DOCX)

S13 Table Characteristics of participants, stratified by colorectal cancer status. (DOCX)
