# Supplementary material for: Genetic variants and traits related to insulin-like growth factor-I and insulin resistance and their interaction with lifestyles on postmenopausal colorectal cancer risk
Source: PLoS One. 2017 Oct 12;12(10):e0186296. doi: 10.1371/journal.pone.0186296 (PMC5638514; doi:10.1371/journal.pone.0186296)
Supplement: S1 Fig — (DOCX) [file pone.0186296.s001.docx]

Figure S1. Flow diagram of analytic cohort.

WHI ancillary studies (AS129 &AS152) participants

(n = 1,136, case = 408, controls = 728)

Excluded women (n = 193) who had been followed up for less than 1 year or were diagnosed with any cancer at enrollment

943 women

Excluded women (n = 55) who had diabetes at baseline

Applied exclusion criteria

888 women

Excluded women (n = 1) whose fasting time for serum biomarkers at baseline was less than 8 hours

887 women

Excluded women (n = 2) without any of 75 SNPs’ information or with more than 50% missing call

885 women (case = 311, control = 574)

Excluded women (n = 181) for whom information on covariates was not available

Included in analysis

704 women (80% of the 885; case = 237, control = 467)
