# Supplementary material for: Genetic variants and traits related to insulin-like growth factor-I and insulin resistance and their interaction with lifestyles on postmenopausal colorectal cancer risk
Source: PLoS One. 2017 Oct 12;12(10):e0186296. doi: 10.1371/journal.pone.0186296 (PMC5638514; doi:10.1371/journal.pone.0186296)
Supplement: S1 Table — (DOCX) [file pone.0186296.s002.docx]

Table S1. Allele frequencies of 33 IGF-I/insulin pathways–relevant SNPs, stratified by obesity (measured via BMI)

| **SNP** | **Chromosome** | **Allele**  **(effect/baseline)** | **Effect allele frequency** | | |
| --- | --- | --- | --- | --- | --- |
|  |  |  | **Non-obese group**  **(BMI < 30.0 kg/m^2^)** |  | **Obese group**  **(BMI ≥ 30.0 kg/m^2^)** |
|  |  |  | **(n = 527)** |  | **(n = 177)** |
| **IGF1RS10745942** | 12 | A/C | 6.9 |  | 6.8 |
| **IGF1RS10778176** | 12 | T/C | 27.1 |  | 29.5 |
| **IGF1RS10860865** | 12 | T/G | 27.0 |  | 29.0 |
| **IGF1RS1520220** | 12 | G/C | 19.1 |  | 20.1 |
| **IGF1RS35767** | 12 | T/C | 16.3 |  | 16.4 |
| **IGF1RS5742612** | 12 | G/A | 3.8 |  | 4.0 |
| **IGF1RS5742671** | 12 | A/G | 18.5 |  | 21.2 |
| **IGF1RS6214** | 12 | A/G | 38.3 |  | 42.1 |
| **IGF1RS6219** | 12 | A/G | 10.2 |  | 11.9 |
| **IGF1RS7136446** | 12 | C/T | 40.0 |  | 41.2 |
| **IGF1RS978458** | 12 | T/C | 26.7 |  | 28.6 |
| **IGFBP3RS1117457** | 7 | A/G | 45.1 |  | 44.9 |
| **IGFBP3RS2132570** | 7 | A/C | 20.8 |  | 21.5 |
| **IGFBP3RS2471551** | 7 | C/G | 21.3 |  | 19.2 |
| **IGFBP3RS3110697** | 7 | A/G | 41.2 |  | 41.7 |
| **IGFBP3RS6670** | 7 | A/T | 22.4 |  | 21.6 |
| **INSRS3842763** | 11 | A/C | 27.0 |  | 25.4 |
| **INSRS3842767** | 11 | A/G | 9.6 |  | 10.2 |
| **INSRS689** | 11 | T/A | 28.8 |  | 28.5 |
| **IRS1RS1801123** | 2 | G/A | 11.2 |  | 11.0 |
| **IRS1RS1801278** | 2 | T/C | 5.7 |  | 6.3 |
| **AKT1RS1130214** | 14 | T/G | 32.4 |  | 26.7 |
| **AKT1RS2494738** | 14 | T/C | 6.2 |  | 7.2 |
| **AKT1RS2494740** | 14 | T/A | 31.9 |  | 32.6 |
| **AKT1RS2494744** | 14 | T/C | 7.0 |  | 8.6 |
| **AKT1RS2498789** | 14 | C/T | 9.5 |  | 10.6 |
| **AKT1RS3001371** | 14 | A/G | 30.8 |  | 30.5 |
| **AKT1RS3803304** | 14 | C/G | 27.2 |  | 24.7 |
| **AKT2RS11673367** | 19 | A/T | 23.8 |  | 23.7 |
| **AKT2RS2304186** | 19 | A/C | 44.1 |  | 45.1 |
| **AKT2RS3730256** | 19 | T/C | 10.2 |  | 8.3 |
| **AKT2RS4332845** | 19 | A/T | 32.6 |  | 32.4 |
| **AKT2RS7247515** | 19 | A/G | 7.6 |  | 4.6 |

BMI, body mass index; IGF-I, insulin-like growth factor-I; SNP, single-nucleotide polymorphism.
