# Supplementary material for: Genetic variants and traits related to insulin-like growth factor-I and insulin resistance and their interaction with lifestyles on postmenopausal colorectal cancer risk
Source: PLoS One. 2017 Oct 12;12(10):e0186296. doi: 10.1371/journal.pone.0186296 (PMC5638514; doi:10.1371/journal.pone.0186296)
Supplement: S2 Table — (DOCX) [file pone.0186296.s003.docx]

Table S2. Allele frequencies of 33 IGF-I/insulin pathways–relevant SNPs, stratified by obesity (measured via waist circumference)

| **SNP** | **Chromosome** | **Allele**  **(effect/baseline)** | **Effect allele frequency** | | |
| --- | --- | --- | --- | --- | --- |
|  |  |  | **Non-obese group**  **(Waist ≤ 88 cm)** |  | **Obese group**  **(Waist > 88 cm)** |
|  |  |  | **(n = 456)** |  | **(n = 248)** |
| **IGF1RS10745942** | 12 | A/C | 6.7 |  | 7.1 |
| **IGF1RS10778176** | 12 | T/C | 27.1 |  | 28.7 |
| **IGF1RS10860865** | 12 | T/G | 26.4 |  | 29.5 |
| **IGF1RS1520220** | 12 | G/C | 18.6 |  | 20.6 |
| **IGF1RS35767** | 12 | T/C | 16.2 |  | 16.5 |
| **IGF1RS5742612** | 12 | G/A | 4.1 |  | 3.5 |
| **IGF1RS5742671** | 12 | A/G | 18.1 |  | 21.2 |
| **IGF1RS6214** | 12 | A/G | 39.1 |  | 39.5 |
| **IGF1RS6219** | 12 | A/G | 10.0 |  | 11.7 |
| **IGF1RS7136446** | 12 | C/T | 39.4 |  | 42.1 |
| **IGF1RS978458** | 12 | T/C | 25.9 |  | 29.6 |
| **IGFBP3RS1117457** | 7 | A/G | 43.9 |  | 47.2 |
| **IGFBP3RS2132570** | 7 | A/C | 20.8 |  | 21.4 |
| **IGFBP3RS2471551** | 7 | C/G | 21.4 |  | 19.6 |
| **IGFBP3RS3110697** | 7 | A/G | 41.7 |  | 40.7 |
| **IGFBP3RS6670** | 7 | A/T | 23.0 |  | 20.8 |
| **INSRS3842763** | 11 | A/C | 27.9 |  | 24.2 |
| **INSRS3842767** | 11 | A/G | 8.7 |  | 11.7 |
| **INSRS689** | 11 | T/A | 29.2 |  | 28.0 |
| **IRS1RS1801123** | 2 | G/A | 11.5 |  | 10.5 |
| **IRS1RS1801278** | 2 | T/C | 6.1 |  | 5.5 |
| **AKT1RS1130214** | 14 | T/G | 31.4 |  | 30.1 |
| **AKT1RS2494738** | 14 | T/C | 5.8 |  | 7.6 |
| **AKT1RS2494740** | 14 | T/A | 30.9 |  | 34.1 |
| **AKT1RS2494744** | 14 | T/C | 6.4 |  | 9.1 |
| **AKT1RS2498789** | 14 | C/T | 9.4 |  | 10.6 |
| **AKT1RS3001371** | 14 | A/G | 29.7 |  | 32.6 |
| **AKT1RS3803304** | 14 | C/G | 26.5 |  | 26.7 |
| **AKT2RS11673367** | 19 | A/T | 24.5 |  | 22.4 |
| **AKT2RS2304186** | 19 | A/C | 44.0 |  | 45.1 |
| **AKT2RS3730256** | 19 | T/C | 10.2 |  | 8.9 |
| **AKT2RS4332845** | 19 | A/T | 32.2 |  | 33.2 |
| **AKT2RS7247515** | 19 | A/G | 7.5 |  | 5.7 |

IGF-I, insulin-like growth factor-I; SNP, single-nucleotide polymorphism.
