# Supplementary material for: Genetic variants and traits related to insulin-like growth factor-I and insulin resistance and their interaction with lifestyles on postmenopausal colorectal cancer risk
Source: PLoS One. 2017 Oct 12;12(10):e0186296. doi: 10.1371/journal.pone.0186296 (PMC5638514; doi:10.1371/journal.pone.0186296)
Supplement: S4 Table — (DOCX) [file pone.0186296.s005.docx]

Table S4. Allele frequencies of 33 IGF-I/insulin pathways–relevant SNPs, stratified by physical activity level

| **SNP** | **Chromosome** | **Allele**  **(effect/baseline)** | **Effect allele frequency** | | |
| --- | --- | --- | --- | --- | --- |
|  |  |  | **High physical activity group**  **(MET ≥ 10)** |  | **Low physical activity group**  **(MET < 10)** |
|  |  |  | **(n = 356)** |  | **(n = 348)** |
| **IGF1RS10745942** | 12 | A/C | 7.9 |  | 5.8 |
| **IGF1RS10778176** | 12 | T/C | 27.9 |  | 27.5 |
| **IGF1RS10860865** | 12 | T/G | 28.8 |  | 26.2 |
| **IGF1RS1520220** | 12 | G/C | 19.9 |  | 18.7 |
| **IGF1RS35767** | 12 | T/C | 17.1 |  | 15.4 |
| **IGF1RS5742612** | 12 | G/A | 3.0 |  | 4.8 |
| **IGF1RS5742671** | 12 | A/G | 19.7 |  | 18.7 |
| **IGF1RS6214** | 12 | A/G | 40.0 |  | 38.4 |
| **IGF1RS6219** | 12 | A/G | 10.7 |  | 10.5 |
| **IGF1RS7136446** | 12 | C/T | 42.0 |  | 38.6 |
| **IGF1RS978458** | 12 | T/C | 28.6 |  | 25.8 |
| **IGFBP3RS1117457** | 7 | A/G | 45.8 |  | 44.3 |
| **IGFBP3RS2132570** | 7 | A/C | 21.1 |  | 20.9 |
| **IGFBP3RS2471551** | 7 | C/G | 20.5 |  | 21.0 |
| **IGFBP3RS3110697** | 7 | A/G | 40.8 |  | 42.0 |
| **IGFBP3RS6670** | 7 | A/T | 21.7 |  | 22.8 |
| **INSRS3842763** | 11 | A/C | 29.1 |  | 24.0 |
| **INSRS3842767** | 11 | A/G | 9.8 |  | 9.7 |
| **INSRS689** | 11 | T/A | 29.4 |  | 28.2 |
| **IRS1RS1801123** | 2 | G/A | 11.5 |  | 10.8 |
| **IRS1RS1801278** | 2 | T/C | 6.1 |  | 5.7 |
| **AKT1RS1130214** | 14 | T/G | 29.7 |  | 32.2 |
| **AKT1RS2494738** | 14 | T/C | 6.5 |  | 6.4 |
| **AKT1RS2494740** | 14 | T/A | 31.2 |  | 32.9 |
| **AKT1RS2494744** | 14 | T/C | 7.9 |  | 6.9 |
| **AKT1RS2498789** | 14 | C/T | 10.8 |  | 8.8 |
| **AKT1RS3001371** | 14 | A/G | 29.9 |  | 31.6 |
| **AKT1RS3803304** | 14 | C/G | 25.9 |  | 27.3 |
| **AKT2RS11673367** | 19 | A/T | 23.5 |  | 24.0 |
| **AKT2RS2304186** | 19 | A/C | 44.7 |  | 44.0 |
| **AKT2RS3730256** | 19 | T/C | 8.9 |  | 10.6 |
| **AKT2RS4332845** | 19 | A/T | 32.3 |  | 32.8 |
| **AKT2RS7247515** | 19 | A/G | 6.5 |  | 7.2 |

IGF-I, insulin-like growth factor-I; MET, metabolic equivalent; SNP, single-nucleotide polymorphism.
