# Supplementary material for: Genetic variants and traits related to insulin-like growth factor-I and insulin resistance and their interaction with lifestyles on postmenopausal colorectal cancer risk
Source: PLoS One. 2017 Oct 12;12(10):e0186296. doi: 10.1371/journal.pone.0186296 (PMC5638514; doi:10.1371/journal.pone.0186296)
Supplement: S5 Table — (DOCX) [file pone.0186296.s006.docx]

Table S5. Allele frequencies of 33 IGF-I/insulin pathways–relevant SNPs, stratified by exogenous estrogen use (nonusers vs. E-only users)

| **SNP** | **Chromosome** | **Allele**  **(effect/baseline)** | **Effect allele frequency** | | |
| --- | --- | --- | --- | --- | --- |
|  |  |  | **Non users** |  | **E**-**only users** |
|  |  |  | **(n = 273)** |  | **(n = 205)** |
| **IGF1RS10745942** | 12 | A/C | 6.6 |  | 7.1 |
| **IGF1RS10778176** | 12 | T/C | 26.8 |  | 28.8 |
| **IGF1RS10860865** | 12 | T/G | 26.0 |  | 30.6 |
| **IGF1RS1520220** | 12 | G/C | 17.8 |  | 21.7 |
| **IGF1RS35767** | 12 | T/C | 16.1 |  | 16.3 |
| **IGF1RS5742612** | 12 | G/A | 4.0 |  | 3.9 |
| **IGF1RS5742671** | 12 | A/G | 18.1 |  | 21.2 |
| **IGF1RS6214** | 12 | A/G | 37.3 |  | 40.5 |
| **IGF1RS6219** | 12 | A/G | 9.2 |  | 12.4 |
| **IGF1RS7136446** | 12 | C/T | 37.9 |  | 41.0 |
| **IGF1RS978458** | 12 | T/C | 25.7 |  | 29.9 |
| **IGFBP3RS1117457** | 7 | A/G | 42.7 |  | 45.6 |
| **IGFBP3RS2132570** | 7 | A/C | 20.0 |  | 19.8 |
| **IGFBP3RS2471551** | 7 | C/G | 21.6 |  | 22.0 |
| **IGFBP3RS3110697** | 7 | A/G | 41.6 |  | 41.2 |
| **IGFBP3RS6670** | 7 | A/T | 22.0 |  | 22.1 |
| **INSRS3842763** | 11 | A/C | 27.1 |  | 25.9 |
| **INSRS3842767** | 11 | A/G | 10.8 |  | 9.0 |
| **INSRS689** | 11 | T/A | 26.4 |  | 30.5 |
| **IRS1RS1801123** | 2 | G/A | 12.8 |  | 10.0 |
| **IRS1RS1801278** | 2 | T/C | 5.5 |  | 5.4 |
| **AKT1RS1130214** | 14 | T/G | 29.2 |  | 31.2 |
| **AKT1RS2494738** | 14 | T/C | 6.5 |  | 6.4 |
| **AKT1RS2494740** | 14 | T/A | 32.9 |  | 32.7 |
| **AKT1RS2494744** | 14 | T/C | 9.0 |  | 7.3 |
| **AKT1RS2498789** | 14 | C/T | 11.3 |  | 9.9 |
| **AKT1RS3001371** | 14 | A/G | 31.5 |  | 30.5 |
| **AKT1RS3803304** | 14 | C/G | 27.1 |  | 26.9 |
| **AKT2RS11673367** | 19 | A/T | 23.5 |  | 23.6 |
| **AKT2RS2304186** | 19 | A/C | 43.8 |  | 43.1 |
| **AKT2RS3730256** | 19 | T/C | 9.2 |  | 10.3 |
| **AKT2RS4332845** | 19 | A/T | 33.5 |  | 33.7 |
| **AKT2RS7247515** | 19 | A/G | 5.7 |  | 8.5 |

E, estrogen; IGF-I, insulin-like growth factor-I; SNP, single-nucleotide polymorphism.
