# Supplementary material for: Genetic variants and traits related to insulin-like growth factor-I and insulin resistance and their interaction with lifestyles on postmenopausal colorectal cancer risk
Source: PLoS One. 2017 Oct 12;12(10):e0186296. doi: 10.1371/journal.pone.0186296 (PMC5638514; doi:10.1371/journal.pone.0186296)
Supplement: S7 Table — (DOCX) [file pone.0186296.s008.docx]

Table S7. Characteristics of participants, stratified by obesity (measured via BMI)

| **Characteristic** | **Non-obese group**  **(BMI < 30.0 kg/m^2^)** | | | |  | **Obese group**  **(BMI ≥ 30.0 kg/m^2^)** | | | |
| --- | --- | --- | --- | --- | --- | --- | --- | --- | --- |
|  | **(n = 527)** | | | |  | **(n = 177)** | | | |
|  | **n** | **(%)** |  |  |  | **n** | **(%)** |  |  |
| **Age in years, median (range)** | 64 | (50–79) | | |  | 63 | (50–79) | | |
| **Education** |  |  |  |  |  |  |  |  |  |
| **≤ High school** | 146 | (27.7) |  |  |  | 60 | (33.9) |  |  |
| **> High school** | 381 | (72.3) |  |  |  | 117 | (66.1) |  |  |
| **Family income** |  |  |  |  |  |  |  |  |  |
| **< $35,000** | 195 | (37.0) |  |  |  | 82 | (46.3)* |  |  |
| **≥ $35,000** | 332 | (63.0) |  |  |  | 95 | (53.7) |  |  |
| **Family history of diabetes mellitus** |  |  |  |  |  |  |  |  |  |
| **No** | 366 | (69.4) |  |  |  | 112 | (63.3) |  |  |
| **Yes** | 161 | (30.6) |  |  |  | 65 | (36.7) |  |  |
| **Family history of colorectal cancer** |  |  |  |  |  |  |  |  |  |
| **No** | 431 | (81.8) |  |  |  | 154 | (87.0) |  |  |
| **Yes** | 96 | (18.2) |  |  |  | 23 | (13.0) |  |  |
| **Heart failure ever** |  |  |  |  |  |  |  |  |  |
| **No** | 519 | (98.5) |  |  |  | 175 | (98.9) |  |  |
| **Yes** | 8 | (1.5) |  |  |  | 2 | (1.1) |  |  |
| **High cholesterol requiring pills ever** |  |  |  |  |  |  |  |  |  |
| **No** | 461 | (87.5) |  |  |  | 156 | (88.1) |  |  |
| **Yes** | 66 | (12.5) |  |  |  | 21 | (11.9) |  |  |
| **Smoking status** |  |  |  |  |  |  |  |  |  |
| **Never** | 280 | (53.1) |  |  |  | 82 | (46.3) |  |  |
| **Past** | 215 | (40.8) |  |  |  | 84 | (47.5) |  |  |
| **Current** | 32 | (6.1) |  |  |  | 11 | (6.2) |  |  |
| **METs·hour·week^-1^¶** |  |  |  |  |  |  |  |  |  |
| **< 10** | 237 | (45.0) |  |  |  | 111 | (62.7)* |  |  |
| **≥ 10** | 290 | (55.0) |  |  |  | 66 | (37.3) |  |  |
| **Dietary alcohol per day in g, median (range)** | 0.5 | (0.0–63.2) | | |  | 0.2 | (0.0–66.3)* | | |
| **Waist circumference in cm, median (range)** | 79.0 | (60.8–107.5) | | |  | 98.0 | (75.0–144.0)* | | |
| **Waist-to-hip ratio, median (range)** | 0.78 | (0.49–1.39) | | |  | 0.84 | (0.62–1.03)* | | |
| **Oral contraceptive use** |  |  |  |  |  |  |  |  |  |
| **Never** | 313 | (59.4) |  |  |  | 110 | (62.1) |  |  |
| **Ever** | 214 | (40.6) |  |  |  | 67 | (37.9) |  |  |
| **History of hysterectomy or oophorectomy** |  |  |  |  |  |  |  |  |  |
| **No** | 320 | (60.7) |  |  |  | 105 | (59.3) |  |  |
| **Yes** | 207 | (39.3) |  |  |  | 72 | (40.7) |  |  |
| **Age at menarche in years, median (range)** | 13 | (≤ 9–≥ 17) | | |  | 12 | (≤ 9–≥ 17)* | | |
| **Age at menopause in years, median (range)** | 50 | (30–69) | | |  | 48 | (30–62)* | | |
| **Pregnancy history** |  |  |  |  |  |  |  |  |  |
| **No** | 54 | (10.2) |  |  |  | 25 | (14.1) |  |  |
| **Yes** | 473 | (89.8) |  |  |  | 152 | (85.9) |  |  |
| **Exogenous estrogen use** |  |  | | |  |  |  | | |
| **Never use** | 189 | (39.0) | | |  | 84 | (48.8)* | | |
| **E-only ever users** | 163 | (33.7) | | |  | 42 | (24.4) | | |
| **E + P ever users** | 132 | (27.3) | | |  | 46 | (26.7) | | |
| **Total IGF-I in ng/mL, median (range)** | 121.2 | (19.3–335.6) | | |  | 120.7 | (35.3–231.8) | | |

Table S7 (Continued)

| **Characteristic** | **Non-obese group**  **(BMI < 30.0 kg/m^2^)** | | | |  | **Obese group**  **(BMI ≥ 30.0 kg/m^2^)** | | | |
| --- | --- | --- | --- | --- | --- | --- | --- | --- | --- |
|  | **(n = 527)** | | | |  | **(n = 177)** | | | |
|  | **n** | **(%)** |  |  |  | **n** | **(%)** |  |  |
| **Free IGF-I in ng/mL, median (range)** | 0.30 | (0.02–2.2) | | |  | 0.34 | (0.02–3.04) | | |
| **IGFBP-3 in ng/mL, median (range)** | 4108 | (1516–7282) | | |  | 4261 | (1536–6183) | | |
| **Glucose in mg/dL, median (range)** | 90.0 | (64.0–179.0) | | |  | 96.0 | (65.0–244.0)* | | |
| **Insulin in μIU/mL, median (range)** | 4.5 | (0.4–119.4) | | |  | 8.7 | (1.7–45.6)* | | |
| **HOMA-IR, median (range)** | 0.99 | (0.09–24.81) | | |  | 2.10 | (0.35–21.3)* | | |

BMI, body mass index; E, estrogen; E+P, estrogen + progestin; HOMA-IR, homeostatic model assessment–insulin resistance; IGF-I, insulin-like growth factor-I; IGFBP-3, IGF binding protein-3; MET, metabolic equivalent.

* *P* < 0.05, chi-squared or Wilcoxon’s rank-sum test.

¶ Physical activity was estimated from recreational physical activity combining walking and mild, moderate, and strenuous physical activity; each activity was assigned a MET value corresponding to intensity, and the total MET·hours·week^-1^ was calculated by multiplying the MET level for the activity by the hours exercised per week and summing the values for all activities. The total MET was stratified into two groups, with 10 METs as the cutoff according to current American College of Sports Medicine and American Heart Association recommendations.(77)
