# Supplementary material for: Genetic variants and traits related to insulin-like growth factor-I and insulin resistance and their interaction with lifestyles on postmenopausal colorectal cancer risk
Source: PLoS One. 2017 Oct 12;12(10):e0186296. doi: 10.1371/journal.pone.0186296 (PMC5638514; doi:10.1371/journal.pone.0186296)
Supplement: S8 Table — (DOCX) [file pone.0186296.s009.docx]

Table S8. Characteristics of participants, stratified by obesity (measured via waist circumference)

| **Characteristic** | **Non-obese group (waist ≤ 88 cm)** | | | |  | **Obese group (waist > 88 cm)** | | | |
| --- | --- | --- | --- | --- | --- | --- | --- | --- | --- |
|  | **(n = 456)** | | | |  | **(n = 248)** | | | |
|  | **n** | **(%)** |  |  |  | **n** | **(%)** |  |  |
| **Age in years, median (range)** | 64 | (50–79) | | |  | 64 | (50–79) | | |
| **Education** |  |  |  |  |  |  |  |  |  |
| **≤ High school** | 120 | (26.3) |  |  |  | 86 | (34.7)* |  |  |
| **> High school** | 336 | (73.7) |  |  |  | 162 | (65.3) |  |  |
| **Family income** |  |  |  |  |  |  |  |  |  |
| **< $35,000** | 157 | (34.4) |  |  |  | 120 | (48.4)* |  |  |
| **≥ $35,000** | 299 | (65.6) |  |  |  | 128 | (51.6) |  |  |
| **Family history of diabetes mellitus** |  |  |  |  |  |  |  |  |  |
| **No** | 325 | (71.3) |  |  |  | 153 | (61.7)* |  |  |
| **Yes** | 131 | (28.7) |  |  |  | 95 | (38.3) |  |  |
| **Family history of colorectal cancer** |  |  |  |  |  |  |  |  |  |
| **No** | 370 | (81.1) |  |  |  | 215 | (86.7) |  |  |
| **Yes** | 86 | (18.9) |  |  |  | 33 | (13.3) |  |  |
| **Heart failure ever** |  |  |  |  |  |  |  |  |  |
| **No** | 449 | (98.5) |  |  |  | 245 | (98.8) |  |  |
| **Yes** | 7 | (1.5) |  |  |  | 3 | (1.2) |  |  |
| **High cholesterol requiring pills ever** |  |  |  |  |  |  |  |  |  |
| **No** | 412 | (90.4) |  |  |  | 205 | (82.7)* |  |  |
| **Yes** | 44 | (9.6) |  |  |  | 43 | (17.3) |  |  |
| **Smoking status** |  |  |  |  |  |  |  |  |  |
| **Never** | 247 | (54.2) |  |  |  | 115 | (46.4) |  |  |
| **Past** | 180 | (39.5) |  |  |  | 119 | (48.0) |  |  |
| **Current** | 29 | (6.4) |  |  |  | 14 | (5.6) |  |  |
| **METs·hour·week^-1^¶** |  |  |  |  |  |  |  |  |  |
| **< 10** | 193 | (42.3) |  |  |  | 155 | (62.5)* |  |  |
| **≥ 10** | 263 | (57.7) |  |  |  | 93 | (37.5) |  |  |
| **Dietary alcohol per day in g, median (range)** | 0.5 | (0.0–66.3) | | |  | 0.4 | (0.0–31.5)* | | |
| **BMI, kg/m^2^, median (range)** | 24.5 | (15.5–44.9) | | |  | 31.2 | (22.0–59.8)* | | |
| **Waist-to-hip ratio, median (range)** | 0.77 | (0.62–0.99) | | |  | 0.86 | (0.49–1.39)* | | |
| **Oral contraceptive use** |  |  |  |  |  |  |  |  |  |
| **Never** | 270 | (59.2) |  |  |  | 153 | (61.7) |  |  |
| **Ever** | 186 | (40.8) |  |  |  | 95 | (38.3) |  |  |
| **History of hysterectomy or oophorectomy** |  |  |  |  |  |  |  |  |  |
| **No** | 274 | (60.1) |  |  |  | 151 | (60.9) |  |  |
| **Yes** | 182 | (39.9) |  |  |  | 97 | (39.1) |  |  |
| **Age at menarche in years, median (range)** | 13 | (≤ 9–≥ 17) | | |  | 13 | (≤ 9–≥ 17) | | |
| **Age at menopause in years, median (range)** | 49 | (30–69) | | |  | 48 | (30–67) | | |
| **Pregnancy history** |  |  |  |  |  |  |  |  |  |
| **No** | 51 | (11.2) |  |  |  | 28 | (11.3) |  |  |
| **Yes** | 405 | (88.8) |  |  |  | 220 | (88.7) |  |  |
| **Exogenous estrogen use** |  |  | | |  |  |  | | |
| **Never use** | 152 | (36.3) | | |  | 121 | (51.1)* | | |
| **E-only ever users** | 146 | (34.8) | | |  | 59 | (24.9) | | |
| **E + P ever users** | 121 | (28.9) | | |  | 57 | (24.1) | | |

Table S8 (Continued)

| **Characteristic** | **Non-obese group (waist ≤ 88 cm)** | | | |  | **Obese group (waist > 88 cm)** | | | |
| --- | --- | --- | --- | --- | --- | --- | --- | --- | --- |
|  | **(n = 456)** | | | |  | **(n = 248)** | | | |
|  | **n** | **(%)** |  |  |  | **n** | **(%)** |  |  |
| **Total IGF-I in ng/mL, median (range)** | 120.7 | (19.3–335.6) | | |  | 122.1 | (35.3–267.6) | | |
| **Free IGF-I in ng/mL, median (range)** | 0.30 | (0.02–1.95) | | |  | 0.35 | (0.02–3.04) | | |
| **IGFBP-3 in ng/mL, median (range)** | 4054 | (1516–7282) | | |  | 4303 | (1536–6975)* | | |
| **Glucose in mg/dL, median (range)** | 90.0 | (64.0–148.0) | | |  | 95.0 | (65.0–244.0)* | | |
| **Insulin in μIU/mL, median (range)** | 4.1 | (0.4–25.3) | | |  | 8.7 | (1.4–119.4)* | | |
| **HOMA-IR, median (range)** | 0.90 | (0.09–7.17) | | |  | 2.04 | (0.26–24.81)* | | |

BMI, body mass index; E, estrogen; E+P, estrogen + progestin; HOMA-IR, homeostatic model assessment–insulin resistance; IGF-I, insulin-like growth factor-I; IGFBP-3, IGF binding protein-3; MET, metabolic equivalent.

* *P* < 0.05, chi-squared or Wilcoxon’s rank-sum test.

¶ Physical activity was estimated from recreational physical activity combining walking and mild, moderate, and strenuous physical activity.
