# Supplementary material for: Genetic variants and traits related to insulin-like growth factor-I and insulin resistance and their interaction with lifestyles on postmenopausal colorectal cancer risk
Source: PLoS One. 2017 Oct 12;12(10):e0186296. doi: 10.1371/journal.pone.0186296 (PMC5638514; doi:10.1371/journal.pone.0186296)
Supplement: S9 Table — (DOCX) [file pone.0186296.s010.docx]

Table S9. Characteristics of participants, stratified by obesity (measured via w/h ratio)

| **Characteristic** | **Non-obese group (w/h ≤ 0.85)** | | | |  | **Obese group (w/h ≤ 0.85)** | | | |
| --- | --- | --- | --- | --- | --- | --- | --- | --- | --- |
|  | **(n = 533)** | | | |  | **(n = 171)** | | | |
|  | **n** | **(%)** |  |  |  | **n** | **(%)** |  |  |
| **Age in years, median (range)** | 63 | (50–79) | | |  | 66 | (50–79)* | | |
| **Education** |  |  |  |  |  |  |  |  |  |
| **≤ High school** | 148 | (27.8) |  |  |  | 58 | (33.9) |  |  |
| **> High school** | 385 | (72.2) |  |  |  | 113 | (66.1) |  |  |
| **Family income** |  |  |  |  |  |  |  |  |  |
| **< $35,000** | 180 | (33.8) |  |  |  | 97 | (56.7)* |  |  |
| **≥ $35,000** | 353 | (66.2) |  |  |  | 74 | (43.3) |  |  |
| **Family history of diabetes mellitus** |  |  |  |  |  |  |  |  |  |
| **No** | 372 | (69.8) |  |  |  | 106 | (62.0) |  |  |
| **Yes** | 161 | (30.2) |  |  |  | 65 | (38.0) |  |  |
| **Family history of colorectal cancer** |  |  |  |  |  |  |  |  |  |
| **No** | 445 | (83.5) |  |  |  | 140 | (81.9) |  |  |
| **Yes** | 88 | (16.5) |  |  |  | 31 | (18.1) |  |  |
| **Heart failure ever** |  |  |  |  |  |  |  |  |  |
| **No** | 526 | (98.7) |  |  |  | 168 | (98.2) |  |  |
| **Yes** | 7 | (1.3) |  |  |  | 3 | (1.8) |  |  |
| **High cholesterol requiring pills ever** |  |  |  |  |  |  |  |  |  |
| **No** | 479 | (89.9) |  |  |  | 138 | (80.7)* |  |  |
| **Yes** | 54 | (10.1) |  |  |  | 33 | (19.3) |  |  |
| **Smoking status** |  |  |  |  |  |  |  |  |  |
| **Never** | 288 | (54.0) |  |  |  | 74 | (43.3) |  |  |
| **Past** | 214 | (40.2) |  |  |  | 85 | (49.7) |  |  |
| **Current** | 31 | (5.8) |  |  |  | 12 | (7.0) |  |  |
| **METs·hour·week^-1^¶** |  |  |  |  |  |  |  |  |  |
| **< 10** | 241 | (45.2) |  |  |  | 107 | (62.6)* |  |  |
| **≥ 10** | 292 | (54.8) |  |  |  | 64 | (37.4) |  |  |
| **Dietary alcohol per day in g, median (range)** | 0.4 | (0.0–63.2) | | |  | 0.4 | (0.0–66.3) | | |
| **BMI, kg/m^2^, median (range)** | 25.3 | (15.5–59.8) | | |  | 29.8 | (19.2–51.6)* | | |
| **Waist circumference in cm, median (range)** | 79.0 | (60.8–144.0) | | |  | 96.5 | (77.0–140.4)* | | |
| **Oral contraceptive use** |  |  |  |  |  |  |  |  |  |
| **Never** | 313 | (58.7) |  |  |  | 110 | (64.3) |  |  |
| **Ever** | 220 | (41.3) |  |  |  | 61 | (35.7) |  |  |
| **History of hysterectomy or oophorectomy** |  |  |  |  |  |  |  |  |  |
| **No** | 319 | (59.8) |  |  |  | 106 | (62.0) |  |  |
| **Yes** | 214 | (40.2) |  |  |  | 65 | (38.0) |  |  |
| **Age at menarche in years, median (range)** | 13 | (≤ 9–≥ 17) | | |  | 12 | (≤ 9–≥ 17) | | |
| **Age at menopause in years, median (range)** | 49 | (30–69) | | |  | 49 | (31–67) | | |
| **Pregnancy history** |  |  |  |  |  |  |  |  |  |
| **No** | 63 | (11.8) |  |  |  | 16 | (9.4) |  |  |
| **Yes** | 470 | (88.2) |  |  |  | 155 | (90.6) |  |  |
| **Exogenous estrogen use** |  |  | | |  |  |  | | |
| **Never use** | 185 | (37.3) | | |  | 88 | (55.0)* | | |
| **E-only ever users** | 163 | (32.9) | | |  | 42 | (26.2) | | |
| **E + P ever users** | 148 | (29.8) | | |  | 30 | (18.8) | | |
| **Total IGF-I in ng/mL, median (range)** | 119.3 | (19.3–335.6) | | |  | 124.5 | (35.3–276.4) | | |

Table S9 (Continued)

| **Characteristic** | **Non-obese group (w/h ≤ 0.85)** | | | |  | **Obese group (w/h ≤ 0.85)** | | | |
| --- | --- | --- | --- | --- | --- | --- | --- | --- | --- |
|  | **(n = 533)** | | | |  | **(n = 171)** | | | |
|  | **n** | **(%)** |  |  |  | **n** | **(%)** |  |  |
| **Free IGF-I in ng/mL, median (range)** | 0.31 | (0.02–3.04) | | |  | 0.33 | (0.02–2.22) | | |
| **IGFBP-3 in ng/mL, median (range)** | 4132 | (1516–7282) | | |  | 4275 | (1536–6975) | | |
| **Glucose in mg/dL, median (range)** | 90.0 | (64.0–244.0) | | |  | 96.0 | (71.0–211.0)* | | |
| **Insulin in μIU/mL, median (range)** | 4.6 | (0.4–31.6) | | |  | 8.7 | (1.4–119.4)* | | |
| **HOMA-IR, median (range)** | 1.00 | (0.09–8.58) | | |  | 2.09 | (0.26–24.81)* | | |

BMI, body mass index; E, estrogen; E+P, estrogen + progestin; HOMA-IR, homeostatic model assessment–insulin resistance; IGF-I, insulin-like growth factor-I; IGFBP-3, IGF binding protein-3; MET, metabolic equivalent; w/h ratio, waist-to-hip ratio.

* *P* < 0.05, chi-squared or Wilcoxon’s rank-sum test.

¶ Physical activity was estimated from recreational physical activity combining walking and mild, moderate, and strenuous physical activity.
