# Supplementary material for: Genetic variants and traits related to insulin-like growth factor-I and insulin resistance and their interaction with lifestyles on postmenopausal colorectal cancer risk
Source: PLoS One. 2017 Oct 12;12(10):e0186296. doi: 10.1371/journal.pone.0186296 (PMC5638514; doi:10.1371/journal.pone.0186296)
Supplement: S10 Table — (DOCX) [file pone.0186296.s011.docx]

Table S10. Characteristics of participants, stratified by physical activity level

| **Characteristic** | **High physical activity group**  **(MET ≥ 10)** | | | |  | **Low physical activity group**  **(MET < 10)** | | | |
| --- | --- | --- | --- | --- | --- | --- | --- | --- | --- |
|  | **(n = 356)** | | | |  | **(n = 348)** | | | |
|  | **n** | **(%)** |  |  |  | **n** | **(%)** |  |  |
| **Age in years, median (range)** | 64 | (50–79) | | |  | 64 | (50–79) | | |
| **Education** |  |  |  |  |  |  |  |  |  |
| **≤ High school** | 91 | (25.6) |  |  |  | 115 | (33.0)* |  |  |
| **> High school** | 265 | (74.4) |  |  |  | 233 | (67.0) |  |  |
| **Family income** |  |  |  |  |  |  |  |  |  |
| **< $35,000** | 113 | (31.7) |  |  |  | 164 | (47.1)* |  |  |
| **≥ $35,000** | 243 | (68.3) |  |  |  | 184 | (52.9) |  |  |
| **Family history of diabetes mellitus** |  |  |  |  |  |  |  |  |  |
| **No** | 244 | (68.5) |  |  |  | 234 | (67.2) |  |  |
| **Yes** | 112 | (31.5) |  |  |  | 114 | (32.8) |  |  |
| **Family history of colorectal cancer** |  |  |  |  |  |  |  |  |  |
| **No** | 293 | (82.3) |  |  |  | 292 | (83.9) |  |  |
| **Yes** | 63 | (17.7) |  |  |  | 56 | (16.1) |  |  |
| **Heart failure ever** |  |  |  |  |  |  |  |  |  |
| **No** | 351 | (98.6) |  |  |  | 343 | (98.6) |  |  |
| **Yes** | 5 | (1.4) |  |  |  | 5 | (1.4) |  |  |
| **High cholesterol requiring pills ever** |  |  |  |  |  |  |  |  |  |
| **No** | 309 | (86.8) |  |  |  | 308 | (88.5) |  |  |
| **Yes** | 47 | (13.2) |  |  |  | 40 | (11.5) |  |  |
| **Smoking status** |  |  |  |  |  |  |  |  |  |
| **Never** | 183 | (51.4) |  |  |  | 179 | (51.4)* |  |  |
| **Past** | 164 | (46.1) |  |  |  | 135 | (38.8) |  |  |
| **Current** | 9 | (2.5) |  |  |  | 34 | (9.8) |  |  |
| **Dietary alcohol per day in g, median (range)** | 0.7 | (0.0–66.3) | | |  | 0.4 | (0.0–42.1)* | | |
| **BMI, kg/m^2^, median (range)** | 25.6 | (15.5–44.9) | | |  | 27.3 | (16.7–59.8)* | | |
| **Waist circumference in cm, median (range)** | 80.5 | (60.8–121.5) | | |  | 85.9 | (64.0–144.0)* | | |
| **Waist-to-hip ratio, median (range)** | 0.79 | (0.49–1.39) | | |  | 0.81 | (0.62–1.03)* | | |
| **Oral contraceptive use** |  |  |  |  |  |  |  |  |  |
| **Never** | 202 | (56.7) |  |  |  | 221 | (63.5) |  |  |
| **Ever** | 154 | (43.3) |  |  |  | 127 | (36.5) |  |  |
| **History of hysterectomy or oophorectomy** |  |  |  |  |  |  |  |  |  |
| **No** | 212 | (59.6) |  |  |  | 213 | (61.2) |  |  |
| **Yes** | 144 | (40.4) |  |  |  | 135 | (38.8) |  |  |
| **Age at menarche in years, median (range)** | 13 | (≤ 9–≥ 17) | | |  | 13 | (≤ 9–≥ 17) | | |
| **Age at menopause in years, median (range)** | 50 | (30–69) | | |  | 49 | (30–67) | | |
| **Pregnancy history** |  |  |  |  |  |  |  |  |  |
| **No** | 38 | (10.7) |  |  |  | 41 | (11.8) |  |  |
| **Yes** | 318 | (89.3) |  |  |  | 307 | (88.2) |  |  |
| **Exogenous estrogen use** |  |  | | |  |  |  | | |
| **Never use** | 125 | (37.8) | | |  | 148 | (45.5) | | |
| **E-only ever users** | 104 | (31.4) | | |  | 101 | (31.1) | | |
| **E + P ever users** | 102 | (30.8) | | |  | 76 | (23.4) | | |
| **Total IGF-I in ng/mL, median (range)** | 120.9 | (19.3–281.7) | | |  | 121.6 | (35.3–335.6) | | |
| **Free IGF-I in ng/mL, median (range)** | 0.32 | (0.02–3.04) | | |  | 0.30 | (0.02–1.93) | | |

Table S10 (Continued)

| **Characteristic** | **High physical activity group**  **(MET ≥ 10)** | | | |  | **Low physical activity group**  **(MET < 10)** | | | |
| --- | --- | --- | --- | --- | --- | --- | --- | --- | --- |
|  | **(n = 356)** | | | |  | **(n = 348)** | | | |
|  | **n** | **(%)** |  |  |  | **n** | **(%)** |  |  |
| **IGFBP-3 in ng/mL, median (range)** | 4166 | (1926–7282) | | |  | 4132 | (1516–6515) | | |
| **Glucose in mg/dL, median (range)** | 90.0 | (64.0–143.0) | | |  | 92.0 | (71.0–244.0)* | | |
| **Insulin in μIU/mL, median (range)** | 4.6 | (0.4–119.4) | | |  | 6.3 | (0.9–45.6)* | | |
| **HOMA-IR, median (range)** | 0.99 | (0.09–24.81) | | |  | 1.42 | (0.20–21.29)* | | |

BMI, body mass index; E, estrogen; E+P, estrogen + progestin; HOMA-IR, homeostatic model assessment–insulin resistance; IGF-I, insulin-like growth factor-I; IGFBP-3, IGF binding protein-3; MET, metabolic equivalent.

* *P* < 0.05, chi-squared or Wilcoxon’s rank-sum test.

¶ Physical activity was estimated from recreational physical activity combining walking and mild, moderate, and strenuous physical activity.
