# Supplementary material for: Genetic variants and traits related to insulin-like growth factor-I and insulin resistance and their interaction with lifestyles on postmenopausal colorectal cancer risk
Source: PLoS One. 2017 Oct 12;12(10):e0186296. doi: 10.1371/journal.pone.0186296 (PMC5638514; doi:10.1371/journal.pone.0186296)
Supplement: S11 Table — (DOCX) [file pone.0186296.s012.docx]

Table S11. Characteristics of participants, stratified by exogenous estrogen use (nonusers vs. E**-**only users)

| **Characteristic** | **Non users** | | | |  | **E**-**only users** | | | |
| --- | --- | --- | --- | --- | --- | --- | --- | --- | --- |
|  | **(n = 273)** | | | |  | **(n = 205)** | | | |
|  | **n** | **(%)** |  |  |  | **n** | **(%)** |  |  |
| **Age in years, median (range)** | 66 | (50–79) | | |  | 65 | (50–79) | | |
| **Education** |  |  |  |  |  |  |  |  |  |
| **≤ High school** | 89 | (32.6) |  |  |  | 62 | (30.2) |  |  |
| **> High school** | 184 | (67.4) |  |  |  | 143 | (69.8) |  |  |
| **Family income** |  |  |  |  |  |  |  |  |  |
| **< $35,000** | 132 | (48.4) |  |  |  | 83 | (40.5) |  |  |
| **≥ $35,000** | 141 | (51.6) |  |  |  | 122 | (59.5) |  |  |
| **Family history of diabetes mellitus** |  |  |  |  |  |  |  |  |  |
| **No** | 187 | (68.5) |  |  |  | 142 | (69.3) |  |  |
| **Yes** | 86 | (31.5) |  |  |  | 63 | (30.7) |  |  |
| **Family history of colorectal cancer** |  |  |  |  |  |  |  |  |  |
| **No** | 223 | (81.7) |  |  |  | 172 | (83.9) |  |  |
| **Yes** | 50 | (18.3) |  |  |  | 33 | (16.1) |  |  |
| **Heart failure ever** |  |  |  |  |  |  |  |  |  |
| **No** | 268 | (98.2) |  |  |  | 201 | (98.0) |  |  |
| **Yes** | 5 | (1.8) |  |  |  | 4 | (2.0) |  |  |
| **High cholesterol requiring pills ever** |  |  |  |  |  |  |  |  |  |
| **No** | 234 | (85.7) |  |  |  | 181 | (88.3) |  |  |
| **Yes** | 39 | (14.3) |  |  |  | 24 | (11.7) |  |  |
| **Smoking status** |  |  |  |  |  |  |  |  |  |
| **Never** | 149 | (54.6) |  |  |  | 102 | (49.8) |  |  |
| **Past** | 107 | (39.2) |  |  |  | 93 | (45.4) |  |  |
| **Current** | 17 | (6.2) |  |  |  | 10 | (4.9) |  |  |
| **METs·hour·week^-1^¶** |  |  |  |  |  |  |  |  |  |
| **< 10** | 148 | (54.2) |  |  |  | 101 | (49.3) |  |  |
| **≥ 10** | 125 | (45.8) |  |  |  | 104 | (50.7) |  |  |
| **Dietary alcohol per day in g, median (range)** | 0.4 | (0.0–66.3) | | |  | 0.4 | (0.0–63.2) | | |
| **BMI, kg/m^2^, median (range)** | 27.0 | (15.5–51.6) | | |  | 26.1 | (17.5–53.4)* | | |
| **Waist circumference in cm, median (range)** | 85.0 | (60.8–140.4) | | |  | 82.5 | (62.0–134.5)* | | |
| **Waist-to-hip ratio, median (range)** | 0.81 | (0.49–1.39) | | |  | 0.80 | (0.62–1.03) | | |
| **Oral contraceptive use** |  |  |  |  |  |  |  |  |  |
| **Never** | 198 | (72.5) |  |  |  | 112 | (54.6)* |  |  |
| **Ever** | 75 | (27.5) |  |  |  | 93 | (45.4) |  |  |
| **History of hysterectomy or oophorectomy** |  |  |  |  |  |  |  |  |  |
| **No** | 201 | (73.6) |  |  |  | 32 | (15.6)* |  |  |
| **Yes** | 72 | (26.4) |  |  |  | 173 | (84.4) |  |  |
| **Age at menarche in years, median (range)** | 13 | (≤ 9–≥ 17) | | |  | 13 | (≤ 9–≥ 17) | | |
| **Age at menopause in years, median (range)** | 50 | (30–63) | | |  | 45 | (30–69)* | | |
| **Pregnancy history** |  |  |  |  |  |  |  |  |  |
| **No** | 35 | (12.8) |  |  |  | 19 | (9.3) |  |  |
| **Yes** | 238 | (87.2) |  |  |  | 186 | (90.7) |  |  |
| **Total IGF-I in ng/mL, median (range)** | 138.6 | (45.4–279.5) | | |  | 110.2 | (32.9–281.7)* | | |
| **Free IGF-I in ng/mL, median (range)** | 0.38 | (0.03–2.14) | | |  | 0.28 | (0.02–3.04)* | | |
| **IGFBP-3 in ng/mL, median (range)** | 4237 | (2208–6518) | | |  | 3986 | (1536–7282)* | | |

Table S11 (Continued)

| **Characteristic** | **Non users** | | | |  | **E**-**only users** | | | |
| --- | --- | --- | --- | --- | --- | --- | --- | --- | --- |
|  | **(n = 273)** | | | |  | **(n = 205)** | | | |
|  | **n** | **(%)** |  |  |  | **n** | **(%)** |  |  |
| **Glucose in mg/dL, median (range)** | 93.0 | (75.0–179.0) | | |  | 90.0 | (67.0–154.0)* | | |
| **Insulin in μIU/mL, median (range)** | 5.7 | (1.0–31.6) | | |  | 5.1 | (0.4–119.4) | | |
| **HOMA-IR, median (range)** | 1.32 | (0.23–8.75) | | |  | 1.16 | (0.09–24.81) | | |

BMI, body mass index; E, estrogen; HOMA-IR, homeostatic model assessment–insulin resistance; IGF-I, insulin-like growth factor-I; IGFBP-3, IGF binding protein-3; MET, metabolic equivalent.

* *P* < 0.05, chi-squared or Wilcoxon’s rank-sum test.

¶ Physical activity was estimated from recreational physical activity combining walking and mild, moderate, and strenuous physical activity.
