# Supplementary material for: Genetic variants and traits related to insulin-like growth factor-I and insulin resistance and their interaction with lifestyles on postmenopausal colorectal cancer risk
Source: PLoS One. 2017 Oct 12;12(10):e0186296. doi: 10.1371/journal.pone.0186296 (PMC5638514; doi:10.1371/journal.pone.0186296)
Supplement: S12 Table — (DOCX) [file pone.0186296.s013.docx]

Table S12. Characteristics of participants, stratified by exogenous estrogen use (nonusers vs. E+P users)

| **Characteristic** | **Non users** | | | |  | **E+P users** | | | |
| --- | --- | --- | --- | --- | --- | --- | --- | --- | --- |
|  | **(n = 273)** | | | |  | **(n = 178)** | | | |
|  | **n** | **(%)** |  |  |  | **n** | **(%)** |  |  |
| **Age in years, median (range)** | 66 | (50–79) | | |  | 61 | (50–79)* | | |
| **Education** |  |  |  |  |  |  |  |  |  |
| **≤ High school** | 89 | (32.6) |  |  |  | 47 | (26.4) |  |  |
| **> High school** | 184 | (67.4) |  |  |  | 131 | (73.6) |  |  |
| **Family income** |  |  |  |  |  |  |  |  |  |
| **< $35,000** | 132 | (48.4) |  |  |  | 49 | (27.5)* |  |  |
| **≥ $35,000** | 141 | (51.6) |  |  |  | 129 | (72.5) |  |  |
| **Family history of diabetes mellitus** |  |  |  |  |  |  |  |  |  |
| **No** | 187 | (68.5) |  |  |  | 120 | (67.4) |  |  |
| **Yes** | 86 | (31.5) |  |  |  | 58 | (32.6) |  |  |
| **Family history of colorectal cancer** |  |  |  |  |  |  |  |  |  |
| **No** | 223 | (81.7) |  |  |  | 150 | (84.3) |  |  |
| **Yes** | 50 | (18.3) |  |  |  | 28 | (15.7) |  |  |
| **Heart failure ever** |  |  |  |  |  |  |  |  |  |
| **No** | 268 | (98.2) |  |  |  | 178 | (100) |  |  |
| **Yes** | 5 | (1.8) |  |  |  | 0 | (0) |  |  |
| **High cholesterol requiring pills ever** |  |  |  |  |  |  |  |  |  |
| **No** | 234 | (85.7) |  |  |  | 163 | (91.6) |  |  |
| **Yes** | 39 | (14.3) |  |  |  | 15 | (8.4) |  |  |
| **Smoking status** |  |  |  |  |  |  |  |  |  |
| **Never** | 149 | (54.6) |  |  |  | 89 | (50.0) |  |  |
| **Past** | 107 | (39.2) |  |  |  | 78 | (43.8) |  |  |
| **Current** | 17 | (6.2) |  |  |  | 11 | (6.2) |  |  |
| **METs·hour·week^-1^¶** |  |  |  |  |  |  |  |  |  |
| **< 10** | 148 | (54.2) |  |  |  | 76 | (42.7)* |  |  |
| **≥ 10** | 125 | (45.8) |  |  |  | 102 | (57.3) |  |  |
| **Dietary alcohol per day in g, median (range)** | 0.4 | (0.0–66.3) | | |  | 0.8 | (0.0–30.1)* | | |
| **BMI, kg/m^2^, median (range)** | 27.0 | (15.5–51.6) | | |  | 26.1 | (16.7–59.8)* | | |
| **Waist circumference in cm, median (range)** | 85.0 | (60.8–140.4) | | |  | 80.6 | (62.0–144.0)* | | |
| **Waist-to-hip ratio, median (range)** | 0.81 | (0.49–1.39) | | |  | 0.78 | (0.63–1.00)* | | |
| **Oral contraceptive use** |  |  |  |  |  |  |  |  |  |
| **Never** | 198 | (72.5) |  |  |  | 82 | (46.1)* |  |  |
| **Ever** | 75 | (27.5) |  |  |  | 96 | (53.9) |  |  |
| **History of hysterectomy or oophorectomy** |  |  |  |  |  |  |  |  |  |
| **No** | 201 | (73.6) |  |  |  | 165 | (92.7)* |  |  |
| **Yes** | 72 | (26.4) |  |  |  | 13 | (7.3) |  |  |
| **Age at menarche in years, median (range)** | 13 | (≤ 9–≥ 17) | | |  | 13 | (≤ 9–≥ 17) | | |
| **Age at menopause in years, median (range)** | 50 | (30–63) | | |  | 50 | (36–67)* | | |
| **Pregnancy history** |  |  |  |  |  |  |  |  |  |
| **No** | 35 | (12.8) |  |  |  | 19 | (10.7) |  |  |
| **Yes** | 238 | (87.2) |  |  |  | 159 | (89.3) |  |  |
| **Total IGF-I in ng/mL, median (range)** | 138.6 | (45.4–279.5) | | |  | 115.1 | (19.3–335.6)* | | |
| **Free IGF-I in ng/mL, median (range)** | 0.38 | (0.03–2.14) | | |  | 0.25 | (0.02–1.95)* | | |
| **IGFBP-3 in ng/mL, median (range)** | 4237 | (2208–6518) | | |  | 4138 | (1516–6975) | | |

Table S12 (Continued)

| **Characteristic** | **Non users** | | | |  | **E+P users** | | | |
| --- | --- | --- | --- | --- | --- | --- | --- | --- | --- |
|  | **(n = 273)** | | | |  | **(n = 178)** | | | |
|  | **n** | **(%)** |  |  |  | **n** | **(%)** |  |  |
| **Glucose in mg/dL, median (range)** | 93.0 | (75.0–179.0) | | |  | 90.0 | (64.0–244.0)* | | |
| **Insulin in μIU/mL, median (range)** | 5.7 | (1.0–31.6) | | |  | 4.9 | (0.5–45.6)* | | |
| **HOMA-IR, median (range)** | 1.32 | (0.23–8.75) | | |  | 1.08 | (0.10–21.29)* | | |

BMI, body mass index; E+P, estrogen + progestin; HOMA-IR, homeostatic model assessment–insulin resistance; IGF-I, insulin-like growth factor-I; IGFBP-3, IGF binding protein-3; MET, metabolic equivalent.

* *P* < 0.05, chi-squared or Wilcoxon’s rank-sum test.

¶ Physical activity was estimated from recreational physical activity combining walking and mild, moderate, and strenuous physical activity.
