# Supplementary material for: Genetic variants and traits related to insulin-like growth factor-I and insulin resistance and their interaction with lifestyles on postmenopausal colorectal cancer risk
Source: PLoS One. 2017 Oct 12;12(10):e0186296. doi: 10.1371/journal.pone.0186296 (PMC5638514; doi:10.1371/journal.pone.0186296)
Supplement: S13 Table — (DOCX) [file pone.0186296.s014.docx]

Table S13. Characteristics of participants, stratified by colorectal cancer status

| **Characteristic** | **Controls** | | | |  | **Colorectal cancer cases** | | | |
| --- | --- | --- | --- | --- | --- | --- | --- | --- | --- |
|  | **(n = 467)** | | | |  | **(n = 237)** | | | |
|  | **n** | **(%)** |  |  |  | **n** | **(%)** |  |  |
| **Age in years, median (range)** | 63 | (50–79) | | |  | 66 | (50–79)* | | |
| **Education** |  |  |  |  |  |  |  |  |  |
| **≤ High school** | 137 | (29.3) |  |  |  | 69 | (29.1) |  |  |
| **> High school** | 330 | (70.7) |  |  |  | 168 | (70.9) |  |  |
| **Family income** |  |  |  |  |  |  |  |  |  |
| **< $35,000** | 166 | (35.5) |  |  |  | 111 | (46.8)* |  |  |
| **≥ $35,000** | 301 | (64.5) |  |  |  | 126 | (53.2) |  |  |
| **Family history of diabetes mellitus** |  |  |  |  |  |  |  |  |  |
| **No** | 319 | (68.3) |  |  |  | 159 | (67.1) |  |  |
| **Yes** | 148 | (31.7) |  |  |  | 78 | (32.9) |  |  |
| **Family history of colorectal cancer** |  |  |  |  |  |  |  |  |  |
| **No** | 397 | (85.0) |  |  |  | 188 | (79.3) |  |  |
| **Yes** | 70 | (15.0) |  |  |  | 49 | (20.7) |  |  |
| **Heart failure ever** |  |  |  |  |  |  |  |  |  |
| **No** | 462 | (98.9) |  |  |  | 232 | (97.9) |  |  |
| **Yes** | 5 | (1.1) |  |  |  | 5 | (2.1) |  |  |
| **High cholesterol requiring pills ever** |  |  |  |  |  |  |  |  |  |
| **No** | 412 | (88.2) |  |  |  | 205 | (86.5) |  |  |
| **Yes** | 55 | (11.8) |  |  |  | 32 | (13.5) |  |  |
| **Smoking status** |  |  |  |  |  |  |  |  |  |
| **Never** | 250 | (53.5) |  |  |  | 112 | (47.3) |  |  |
| **Past** | 192 | (41.1) |  |  |  | 107 | (45.1) |  |  |
| **Current** | 25 | (5.4) |  |  |  | 18 | (7.6) |  |  |
| **METs·hour·week^-1^¶** |  |  |  |  |  |  |  |  |  |
| **< 10** | 221 | (47.3) |  |  |  | 127 | (53.6) |  |  |
| **≥ 10** | 246 | (52.7) |  |  |  | 110 | (46.4) |  |  |
| **Dietary alcohol per day in g, median (range)** | 0.4 | (0.0–66.3) | | |  | 0.4 | (0.0–31.5) | | |
| **BMI, kg/m^2^, median (range)** | 26.0 | (16.7–59.8) | | |  | 26.7 | (15.5–45.1)* | | |
| **Waist circumference in cm, median (range)** | 82.0 | (60.8–144.0) | | |  | 85.5 | (64.0–125.0)* | | |
| **Waist-to-hip ratio, median (range)** | 0.79 | (0.62–1.21) | | |  | 0.81 | (0.49–1.39)* | | |
| **Oral contraceptive use** |  |  |  |  |  |  |  |  |  |
| **Never** | 265 | (56.7) |  |  |  | 158 | (66.7)* |  |  |
| **Ever** | 202 | (43.3) |  |  |  | 79 | (33.3) |  |  |
| **History of hysterectomy or oophorectomy** |  |  |  |  |  |  |  |  |  |
| **No** | 273 | (58.5) |  |  |  | 152 | (64.1) |  |  |
| **Yes** | 194 | (41.5) |  |  |  | 85 | (35.9) |  |  |
| **Age at menarche in years, median (range)** | 13 | (≤ 9–≥ 17) | | |  | 13 | (≤ 9–≥ 17) | | |
| **Age at menopause in years, median (range)** | 49 | (30–69) | | |  | 50 | (30–67) | | |
| **Pregnancy history** |  |  |  |  |  |  |  |  |  |
| **No** | 52 | (11.1) |  |  |  | 27 | (11.4) |  |  |
| **Yes** | 415 | (88.9) |  |  |  | 210 | (88.6) |  |  |
| **Exogenous estrogen use** |  |  | | |  |  |  | | |
| **Never use** | 164 | (37.9) | | |  | 109 | (48.9)* | | |
| **E-only ever users** | 142 | (32.8) | | |  | 63 | (28.3) | | |
| **E + P ever users** | 127 | (29.3) | | |  | 51 | (22.9) | | |
| **Total IGF-I in ng/mL, median (range)** | 120.3 | (32.9–276.4) | | |  | 122.7 | (19.3–335.6) | | |

Table S13 (Continued)

| **Characteristic** | **Controls** | | | |  | **Colorectal cancer cases** | | | |
| --- | --- | --- | --- | --- | --- | --- | --- | --- | --- |
|  | **(n = 467)** | | | |  | **(n = 237)** | | | |
|  | **n** | **(%)** |  |  |  | **n** | **(%)** |  |  |
| **Free IGF-I in ng/mL, median (range)** | 0.31 | (0.02–3.04) | | |  | 0.31 | (0.02–2.22) | | |
| **IGFBP-3 in ng/mL, median (range)** | 4135 | (1536–6975) | | |  | 4175 | (1516–7282) | | |
| **Glucose in mg/dL, median (range)** | 91.0 | (64.0–244.0) | | |  | 92.0 | (65.0–191.0)* | | |
| **Insulin in μIU/mL, median (range)** | 4.9 | (0.4–29.6) | | |  | 6.3 | (0.9–119.4)* | | |
| **HOMA-IR, median (range)** | 1.09 | (0.09–8.58) | | |  | 1.45 | (0.20–24.81)* | | |

BMI, body mass index; E, estrogen; E+P, estrogen + progestin; HOMA-IR, homeostatic model assessment–insulin resistance; IGF-I, insulin-like growth factor-I; IGFBP-3, IGF binding protein-3; MET, metabolic equivalent.

* *P* < 0.05, chi-squared or Wilcoxon’s rank-sum test.

¶ Physical activity was estimated from recreational physical activity combining walking and mild, moderate, and strenuous physical activity.
